# Characterization of Regioisomeric Conjugates Arising from Pixantrone at AP Sites in DNA

**DOI:** 10.1021/acs.biochem.6c00116

**Published:** 2026-09-04

**Authors:** Yizhi Fu, John T. Terrell, Andrew Kellum, Pradeep S. Pallan, Rodrigo Galindo-Murillo, Carmelo J. Rizzo, Martin Egli, Michael P. Stone

**Affiliations:** † Department of Chemistry, 5718 Vanderbilt University, Nashville, Tennessee 37235, United States; ‡ Department of Biochemistry, School of Medicine, Vanderbilt University, Nashville, Tennessee 37232, United States; § 2275 Ionis Pharmaceuticals Inc., Carlsbad, California 92010, United States; ∥ Center for Structural Biology, Vanderbilt University, Nashville, Tennessee 37235, United States; ⊥ Vanderbilt-Ingram Cancer Center, Vanderbilt University Medical Center, Nashville, Tennessee 37232, United States

## Abstract

Alkylation at N7 of guanine leads to formation of abasic (AP) sites in DNA. These equilibrate between α and β anomers and form conjugates with 6,9-bis­[(2-aminoethyl)­amino]­benzo­[*g*]­isoquinoline-5,10-dione (Pixantrone, PIX). NMR revealed that these conjugates formed as regioisomeric Schiff base products *R*_a_ and *R*_b_ arising from the nonequivalent PIX C6 and C9 2-aminoethyl side chains. In dsDNA formation of *R*_a_ was favored. In ssDNA, both products formed equally. Structures of reduced *R*_a_ and *R*_b_ conjugates from molecular dynamics calculations restrained by ^1^H NOESY data in 5′-d­(C^1^A^2^G^3^A^4^C^5^T^6^ X ^7^A^8^G^9^A^10^C^11^T^12^)-3′:5′-d­(A^13^G^14^T^15^C^16^T^17^C^18^A^19^G^20^T^21^C^22^T^23^G^24^)-3′, X = reduced PIX-AP site conjugate, showed that both regioisomers intercalated on the 3′ side of base pair T^6^/A^19^, displacing C^18^ into the major groove, with *R*_a_ less intercalated than *R*_b_. The preferential formation of *R*_a_ vs *R*_b_ in dsDNA and deeper intercalation of *R*_b_ may be explained by the differential ability of the asymmetric PIX N2 nitrogen in *R*_a_ vs *R*_b_ to hydrogen bond with the exocyclic *N* ^4^-amine of the cytosine displaced into the major groove. MD simulations in dsDNA with *R*_a_ and *R*_b_ reduced PIX AP site conjugates revealed that for both, the isoquinoline first reoriented within the minor groove. The complementary cytosine rotated toward the major groove, allowing intercalation of the *R*_a_ and *R*_b_ conjugates. Binding energy analyses yielded values of −108.1 (±9.7) kcal/mol and −102.2 (±8.3) kcal/mol for the intercalated *R*_a_ and *R*_b_ conjugates, respectively. A crystal structure in the Dickerson-Drew Dodecamer at 2.5 Å resolution revealed looped-out PIX stacking with PIX from a neighboring duplex.

## Graphical abstract

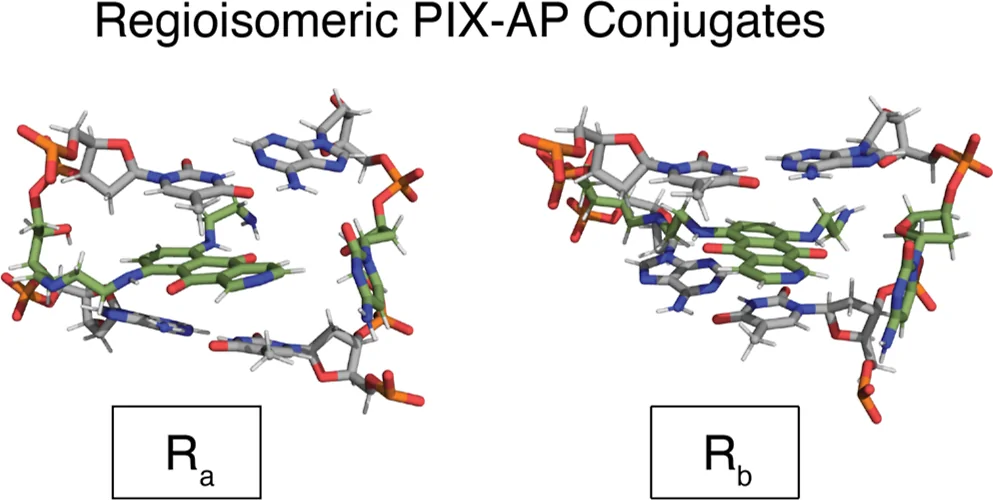

## Introduction

Abasic sites (AP sites) result from spontaneous depurination of canonical and chemically modified nucleobases in DNA^300–1^ but also from DNA alkylation during chemotherapy.^1,2^ They also form during the removal of damaged nucleobases by base excision repair (BER).^3^ Spontaneous depurination generates ∼10^4^ AP sites per cell per day, which are removed by AP endonucleases, with steady-state levels of one AP site per 10^7^ nt.^2,4,302^ Structurally, AP sites retain a B-DNA conformation.^5,6^ However, they are genotoxic, with different dNTPs incorporated opposite these lesions depending on the polymerase and DNA sequence context.^7,8^ AP sites equilibrate between α and β anomers of the 2′-deoxyribosyl sugar via a ring-opened aldehyde,^5,6,9–11^ which places transient levels of an electrophilic aldehyde into the DNA. This renders AP sites in DNA reactive to both endogenous and exogenous amines;^1^ consequently, they form interstrand DNA cross-links,^11–13^ as well as DNA–protein conjugates.^14,15^ Covalent DNA–protein cross-links (DPCs) with repair-associated proteins such as 5-hydroxymethylcytosine binding, embryonic stem-cell-specific (HMCES)^16^ protect the genome by shielding AP sites from endonuclease cleavage and replication-associated processing arising from DNA damage or replication stress.^17^ Bellamri et al.^18^ demonstrated that amino groups of the antitumor agents doxorubicin (DOX), mitoxantrone (MTX), and pixantrone (PIX) (Chart 1) may react with AP sites to form Schiff base conjugates.

**Chart 1:**
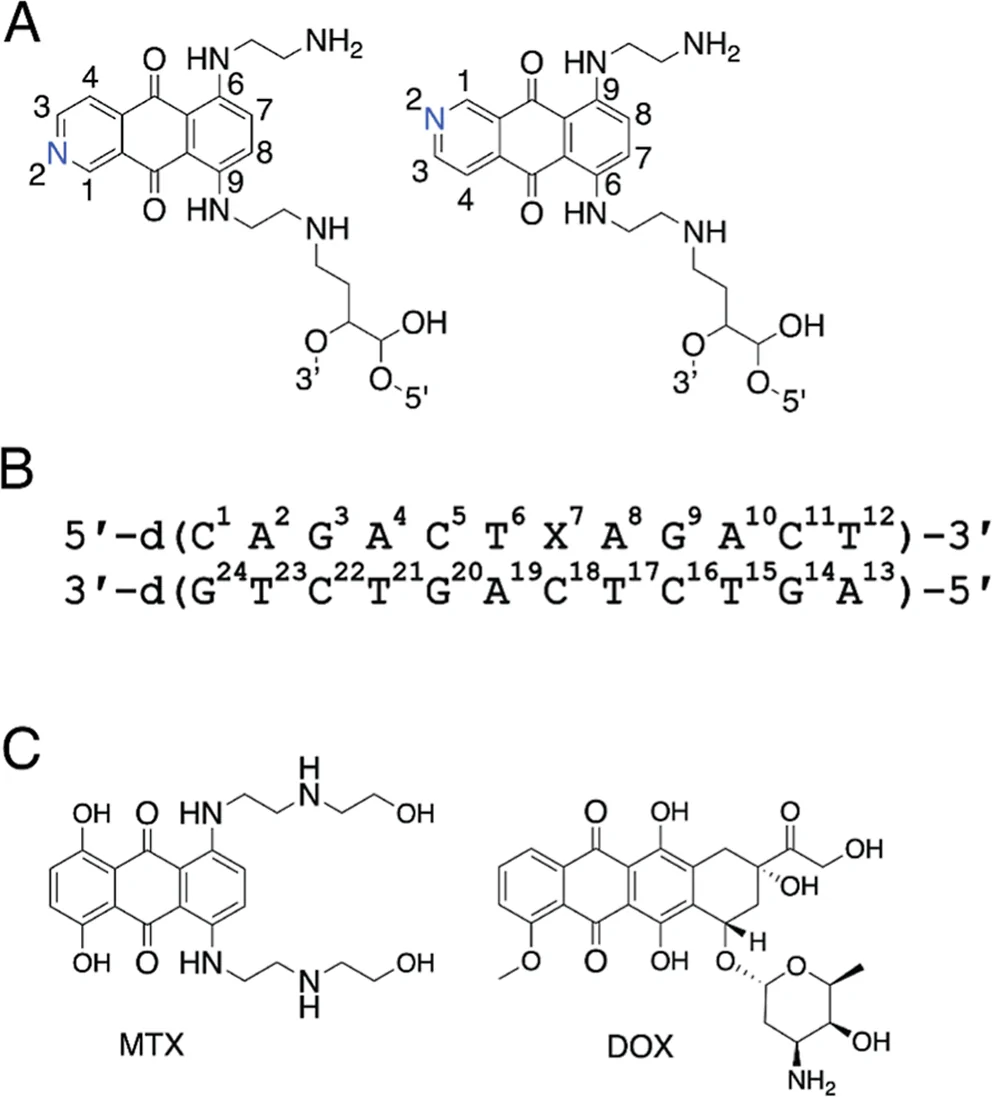
(A) Structures of regioisomeric PIX-AP conjugates, *R*_a_ (left) and *R*_b_ (right). (B) Oligodeoxynucleotide used in this work, showing numbering of individual nucleotides. (C) Structures of mitoxantrone (MTX) and doxorubicin (DOX)

Pixantrone (PIX), 6,9-bis­[(2-aminoethyl)­amino]­benzo­[*g*]­isoquinoline-5,10-dione (CAS Registry 144510-96-3), is a cytotoxic, synthetic aza-anthracenedione antitumor agent.^19^ Its cytotoxicity is believed to involve intercalation into DNA and interference with DNA-topoisomerase II activity during cell division, leading to protein-associated DNA strand breaks and cell death. PIX is a noncardiotoxic^19^ structural analog of MTX, 1,4-dihydroxy-5,8-bis­[2-(2-hydroxyethylamino)­ethylamino]-anthracene-9,10-dione.^20,21^ The aza-anthracenedione ring of PIX is believed to reduce cardiotoxicity by a combination of factors, including its reduced redox cycling ability, reduced ability to bind Fe­(III), and its selectivity for topoisomerase IIa in stabilizing topoisomerase-DNA covalent complexes, as opposed to topoisomerase IIb, expressed in cardiac tissue.^22^ The nitrogen atom in the PIX aza-anthracenedione ring renders the two side-chain amines nonequivalent, and predicts the formation of regioisomeric Schiff base conjugates with AP sites in DNA, referred to as regioisomers A and B and designated as *R*_a_ and *R*_b_ (Chart 1).

Here, we investigate PIX conjugation at AP sites in DNA. We prepared reduced analogs of site-specific PIX-AP site conjugates in oligodeoxynucleotides (Chart 1). We show that PIX forms regioisomeric Schiff base conjugates with AP sites. We identify and characterize these reduced PIX-AP site conjugates using NMR spectroscopy, showing that the regioisomers form equally in ssDNA but at differential levels in dsDNA. We employed restrained molecular dynamics (rMD) simulations to determine structures for the two regioisomers. Both intercalate, displacing the complementary cytosine into the major groove. Calculations and thermal melting studies reveal that both have similar energies. Unbiased molecular dynamics (MD) simulations indicate that PIX intercalates at AP sites from the DNA minor groove, shifting the nonpaired nucleotide in the complementary strand toward the major groove. A possible explanation for the differential distribution of regioisomers is that the reaction proceeds under kinetic control, modulated by differences in pixantrone intercalation geometries involving the nitrogen atom in the PIX aza-anthracenedione ring. Formation of the less favored regioisomer might involve a transient H-bonding interaction between the ligand and the exocyclic amino group of the opposing cytosine, which could hinder Schiff-base formation at the AP site. We also present a crystal structure of PIX-AP conjugates in the Dickerson-Drew Dodecamer (DDD, 5′-d­(CGCXAATTCGCG)-3′, X = PIX-AP) refined to a resolution of 2.5 Å. The structure revealed disruption of the DNA duplex and an extrahelical PIX engaged in a stacking interaction with a second extrahelical PIX from a neighboring DNA duplex.

## Materials and Methods

### Unbiased Molecular Dynamics Simulations

The Dickerson-Drew DNA (DDD) dodecamer duplex [5′-d­(CGCGAATTCGCG)-3′]_2_ was constructed using DS Visualizer,^23^ DNA nucleotides in the two strands were numbered 1 to 12 and 13 to 24. PIX was constructed using GaussView 6 ^24^ and optimized using Gaussian 16 (M02-2*x*/6-31G*).^25^ The DDD was described with the OL15 AMBER force field^26^ and PIX was described by the General Amber Force Field^27^ with charges obtained with the RESP^28^ fitting scheme. The systems were solvated using the optimum point charge water model^29^ within a truncated octahedral periodic cell with a 10 Å buffer. Na^+^ and Cl^–^ ions were added to neutralize the charge and additional ions to reach a final concentration of 200 mM using the Joung–Cheatham parameters.^30^ Topologies were created using the tleap module of AmberTools 21.^31^ Initial relaxation, heating and minimization was performed using the 9-step protocol implemented in the AmberMD tool.^32^ Simulations were calculated using the GPU version of pmemd^33^ at 310 K with a Monte Carlo barostat, within the NTP ensemble. Temperature was regulated using Langevin dynamics (gamma = 0.01). All simulations were computed using three independent replicas, each ∼5 μs. Analysis was performed using CPPTRAJ version V6.15.0.^34,35^

### Oligodeoxynucleotide Synthesis and Purification

The dodecamer 5′-d­(CAGACTUAGACT)-3′, containing a site-specific uracil nucleotide, was obtained commercially (Integrated DNA Technologies; Coralville, Iowa). *Escherichia coli* uracil-DNA glycosylase^3^ (UDG; EC 3.2.2.3; New England Biolabs; Ipswich, MA) (0.25 units/μL) was added to the uracil-containing dodecamer in 10 mM NaCl, 100 mM 4-(2-hydroxyethyl)-1-piperazineethanesulfonic acid (HEPES; pH 7.4) with total volume of 1 mL for 1.5 h at 37 °C. Pixantrone dimaleate (Biosynth; Staad, Switzerland) (100 μM) was added simultaneously with sodium cyanoborohydride (Na­[BH_3_(CN)]) (50 mM). Pixantrone is cytotoxic. Sodium cyanoborohydride is toxic. Both should be handled in a fume hood with appropriate personal protective equipment. The mixture was incubated for 3 h at 37 °C; the dodecamer 5′-dCAGACTXAGACT-3′, X = reduced PIX-AP site conjugate, was purified by reverse-phase HPLC, with a Gemini C_18_ column (4.6 mm internal diameter, 10 mm particle size) at flow rate of 1.5 mL/min. The mobile phase was derived by mixing solvent A, 0.1 M ammonium formate (pH 7), with solvent B, acetonitrile. The gradient was 1% to 10% solvent B over 15 min, followed by 10% to 20% solvent B over 5 min, held isocratic for 5 min, then increased to 80% solvent B over 2 min, held isocratic at 80% solvent B for 3 min, then decreased to 0% solvent B over 2 min. The purified dodecamer containing the reduced PIX-AP site conjugate was lyophilized, dissolved in 1 mL of H_2_O, and annealed with the complementary dodecamer 5′-d­(AGTCTCAGTCTG)-3′. The duplex dodecamer containing the reduced PIX-AP site conjugate was passed over a hydroxyapatite (HAP) column (Econo-Column, 2.5 × 10 cm, Bio-Rad, Inc., Hercules, CA), equilibrated with 200 mM NaH_2_PO_4_ (pH 7). Buffer A contained 100 mM NaCl, 50 μM disodium ethylenediaminetetraacetic acid (Na_2_EDTA), and 10 mM NaH_2_PO_4_ (pH 7); buffer B contained 100 mM NaCl, 50 μM Na_2_EDTA, and 200 mM NaH_2_PO_4_. (pH 7). A linear gradient from 0% to 25% Buffer B was run for 1 h, followed by a linear gradient from 25% to 100% buffer B for 40 min, and the HAP column was maintained at 100% B for 20 min. The dodecamer duplex was desalted by passing through Sephadex G-25 (Sigma-Aldrich, St. Louis, MO) packed into a chromatography column (Econo-Column, 1.5 × 75 cm, Bio-Rad Inc., Hercules, CA). Desalting was carried out at a flow rate of 2.0 mL/min using deionized water. DNA elution was monitored by UV absorbance at 260 nm and salt elution was monitored by conductivity.

For crystallographic studies, an aliquot of UDG was added to a solution containing 50 μM 5′-d­(CGCUAATTCGCG)-3′ in 100 mM KCl and 20 μM Tris–HCl (pH 7.4). The solution was incubated at room temperature for 1 h. Next, a 2-fold molar excess of pixantrone dimaleate (Biosynth Carbosynth) was added. Subsequently, an excess of freshly prepared sodium cyanoborohydride was added and the solution incubated at room temperature for 3 h. The resulting PIX-conjugated DDD 5′-CGCXAATTCGCG-3′ was then purified by reverse phase HPLC.

### Mass Spectrometry

Mass-to-charge ratios (*m*/*z*) of oligodeoxynucleotides were measured using a ultraflexXtreme MALDI-TOF/TDF mass spectrometer (Bruker Instruments, Inc., Billerica, MA). Samples were prepared by mixing 10 μL of 0.5 M of 3-hydroxypicolinic acid (HPA), 5 μL of 0.1 M ammonium hydrogen citrate (AHC), and 2 μL of DNA. Both HPA and AHC solutions were dissolved in 1:1 (v/v) acetonitrile/water solution. Mass spectra were acquired in negative ion mode.

### PIX Conjugation Studies

PIX was incubated for 0.5, 1, and 3 h with the duplex dodecamer 5′-d­(CAGACTUAGACT)-3′:5′-d­(AGTCTCAGTCTG)-3′, containing a site-specific uracil nucleotide, in 1 mM NaCl, 100 mM HEPES (pH 7.4) with total volume of 1 mL, as a function of temperature. For single strand DNA studies, pixantrone was incubated with the dodecamer 5′-d­(CAGACTUAGACT)-3′, containing a site-specific uracil nucleotide, for 0.5, 1, and 3 h in 1 mM NaCl, 100 mM HEPES (pH 7.4) with total volume of 1 mL, as a function of temperature. For both duplex DNA and single-strand DNA studies, pixantrone (100 μM) was added simultaneously with Na­[BH_3_(CN)] (50 mM). In dsDNA, reactions were monitored at 0 °C, 25 °C, 35 °C, and 60 °C. In ssDNA, reactions were monitored at 0 °C, 37 and 60 °C. At 0.5, 1, and 3 h, reactions were quenched using 1 M methoxyamine (MX).^36^ The regioisomeric distributions of reduced AP site conjugates were analyzed by ^1^H NMR by integrating the pixantrone H1 and H3 proton signals (Chart 1).

### Melting Temperature (*T*_m_) Experiments

The absorbances of 5 μM 5′-d­(CAGACTXAGACT)-3′:5′-d­(AGTCTCAGTCTG)-3′ (X = reduced PIX-AP site conjugate, or tetrahydrofuran (THF)), and of the corresponding unmodified duplex were measured in 1 M NaCl, 50 μM Na_2_EDTA, and 10 mM NaH_2_PO_4_ (pH 7), at 260 nm, using a 1 cm path length cuvette. Thermal scans were performed from 5 to 85 °C in 1 °C/min intervals. Melting temperature (*T*_m_) values were determined from first derivative analyses of plots obtained for DNA absorbance at 260 nm as a function of temperature. All experiments were performed on a Cary 100 Bio UV/vis spectrometer equipped with a Peltier thermal cycler (Agilent, Santa Clara, CA, USA).

### NMR Spectroscopy

The 5′-d­(CAGACTXAGACT)-3′:5′-d­(AGTCTCAGTCTG)-3′ (X = reduced PIX-AP site conjugate) and the unmodified duplex 5′-d­(CAGACTGAGACT)-3′:5′-d­(AGTCTCAGTCTG)-3′ were prepared in 100 mM NaCl, 50 μM Na_2_EDTA, and 10 mM NaH_2_PO_4_ (pH 7.0). The concentrations of dodecamer duplexes were 300 μM. The sample volumes were maintained at 250 μL. Data were collected in 5 mm NMR tubes with cryogenic probes (Bruker Biospin, Inc., Billerica, MA). ^1^H COSY^37^ spectra and NOESY^38,39^ spectra of nonexchangeable protons were obtained in buffer containing 99.996% D_2_O at 288 K, while NOESY spectra of exchangeable protons were obtained in buffer containing 95:5 H_2_O/D_2_O at 274 K. NOESY spectra of nonexchangeable protons were collected at 100 ms and 250 ms mixing times, and a relaxation delay of 1.5 s. Spectra were acquired with 2048 complex points in the direct dimension (*t*_2_) and 512 increments in the indirect dimension (*t*_1_). The *t*_1_ dimension was zero-filled to 2048 points, resulting in a final processed matrix of 2048 × 2048 data points. Data were analyzed using TOPSPIN (Bruker Biospin, Billerica, MA, USA) and processed using SPARKY.^40^

### NMR Distance Restraints

The volumes of NOESY cross-peaks were integrated from spectra recorded with mixing time of 250 ms at 288 K. The intrinsic integration error was estimated as half of the volume of the weakest cross-peak. Cross-peaks were assigned error values across five levels based on their characteristics. Well-resolved and nonoverlapping cross-peaks were assigned a 10% error margin. A 20% error was applied to strong, slightly broadened, or moderately overlapped cross-peaks with medium signal-to-noise ratios (S/N). Cross-peaks that were strong but moderately broadened or overlapped were assigned a 30% error. A 40% error was attributed to peaks with weak S/N or slight overlap. Highly broadened cross-peaks near the diagonal or water suppression, or those with moderate S/N and overlap, were assigned a 50% error.^41–43^ Distance restraints were generated using MARDIGRAS.^44^ Additional restraints were applied based on canonical Watson–Crick H-bonding distances for B-DNA base pairs.^45^ Watson–Crick H-bonding restraints were not employed for the T^6^/A^19^ base pair and for the C^18^ base opposite X^7^. Restraints for deoxyribose pseudorotation and phosphodiester backbone torsion angles were based on canonical B-DNA values.^45^ Backbone torsion angles were assigned potential energy wells with a window of ±30°, except for nucleotides T^6^, X^7^, C^18^, and A^19^, which were assigned wider windows of ±60°. Backbone torsion angle restraints were centered on typical B-DNA torsion values: α = −60°, β = 180°, γ = 60°, ε = 195°, and ζ = −105°. Partial charges and bond lengths for regioisomeric PIX-AP conjugates were determined using GAUSSIAN with the B3LYP/6-31G* basis set.^25^

### Restrained Molecular Dynamics Simulations

Starting structures of the regioisomeric reduced PIX-AP site conjugates were constructed using MOE,^46^ where the unmodified duplex containing G^7^ was first generated and subsequently modified to the reduced PIX-AP site conjugate using the Builder tool in AMBER.^31^ Further processing was performed in xLEAP,^47,48^ where partial charges and bond lengths for reduced PIX-AP site conjugates obtained from GAUSSIAN^25^ were incorporated. Parameter and coordinate files (.top and.inp) were generated using the AMBER suite of programs.^31,49^ Restrained molecular dynamics (rMD) calculations were performed using a simulated annealing protocol implemented in AMBER^31^ with the parm99 force field.^50^ Solvation was modeled using the generalized Born model,^51^ with the salt concentration set at 0.1 M. A force constant of 30 kcal mol^–1^ Å^–2^ was applied to all restraints. Initial calculations were carried out over 100 ps for 100,000 steps. The system was heated from 0 to 600 K during the first 3000 steps using a 0.5 ps coupling constant and maintained at 600 K for the remaining steps. NOE-derived distance restraints were compared to calculated intensities from emergent structures using complete relaxation matrix analysis with CORMA.^44^ The 20 structures with the lowest deviations from experimental distance restraints were averaged to generate a refined structure. Structural renderings were created using CHIMERA.^52^

### Crystal Growth

Crystallization trials were performed with the Crystal Screen 1 ^53^ Crystal Screen 2, Natrix 1, Natrix 2, and Nucleic Acid Mini Screen^54^ (Hampton Research, Aliso Viejo, CA). DNA was desalted and prepared at a concentration of 1 mM in DNase- and RNase-free water. The hanging drop vapor diffusion technique was used. Droplets with volumes of 2 μL and composed of a 1:1 mixture of sample and crystallization solution were equilibrated against a 600 μL well solution. A slightly modified condition that resulted in crystals of the native oligonucleotide was used to grow crystals of the dodecamer containing 5Br–C:5′-d­(CGCXAATT­(5-BrC)­GCG)-3′. It consisted of 2.2 M ammonium sulfate, and 2.8% to 3.8% PEG400, in 0.1 M HEPES sodium (pH 7.5). Purple crystals grew within 1 wk.

### Diffraction Data Collection, Phasing and Refinement

Crystals were cryoprotected in a solution consisting of the crystallization condition supplemented with 25% glycerol, mounted in a nylon loop, and frozen in liquid nitrogen. Diffraction data were collected in a cold nitrogen stream on beamline 21-ID-D at LS-CAT, APS (Argonne National Laboratory, Argonne, IL, USA) using a wavelength of 0.9184 Å. Reflections were integrated, scaled, and merged using the program HKL2000.^55^ The structure was phased by Br-SAD using Phenix Autosol^56–303^ and the asymmetric unit of the crystal in space group *P*6_4_22 contains two DNA strands. The experimental map after density modification was used to build 22 of the 24 residues with the program COOT.^58^ The initial model was refined in Refmac 5 ^59^ keeping aside 7% of the reflections to compute R-free.^60^ After three cycles of refinement, 5Br–C and PIX residues were built into the electron density, and refinement was continued in Refmac 5 using a dictionary generated using PRODRG.^61^ Water molecules and several ethylene glycol molecules were added into overlapping positive peaks of Fourier 2Fo-Fc sum and Fo-Fc difference electron densities and accepted on the basis of standard distance criteria. Coordinates and structure factors for the final PIX-DNA model were deposited in the Protein Data Bank (http://www.rcsb.org): PDB ID 7U38.

## Results

### Unbiased PIX-DNA MD Simulations

Unbiased MD simulations were performed to probe PIX binding to the Dickerson-Drew dodecamer (DDD). The starting structure was constructed by adding four PIX molecules at 10 Å of the DDD. Three systems were tested: DNA with an AP site (furanose with no base present), an aldehydic form, and unmodified DNA (control). For each, the representative structure of the most populated cluster is shown in Figure 1. The unmodified dsDNA showed PIX interacting within the minor groove and short-lived preintercalation events, where the PIX aromatic component pushed the paired bases toward the major groove. The simulation in which the dsDNA featured the ring-open aldehydic AP site presented events of the PIX intercalated into the gap that was formed when the unpaired cytosine flipped toward the major groove. Simulations having the AP site in dsDNA showed similar interactions as those seen between DNA and PIX, with PIX interacting from the minor groove. It is possible that with extended sampling time, the AP site would show increased intercalation events missing at this time scale. The structural deviation (RMSD) values of the models showed that the three systems were within ∼2–4 Å to the initial (reference) structure. The per nucleotide fluctuations showed increased values of nucleotides within the AP and aldehydic site that were not observed in the control. Small molecules, especially molecules with aromatic regions, tend to form stacking interactions with the terminal base pairs of short nucleic acid duplexes, which was observed here.

**Figure 1:**
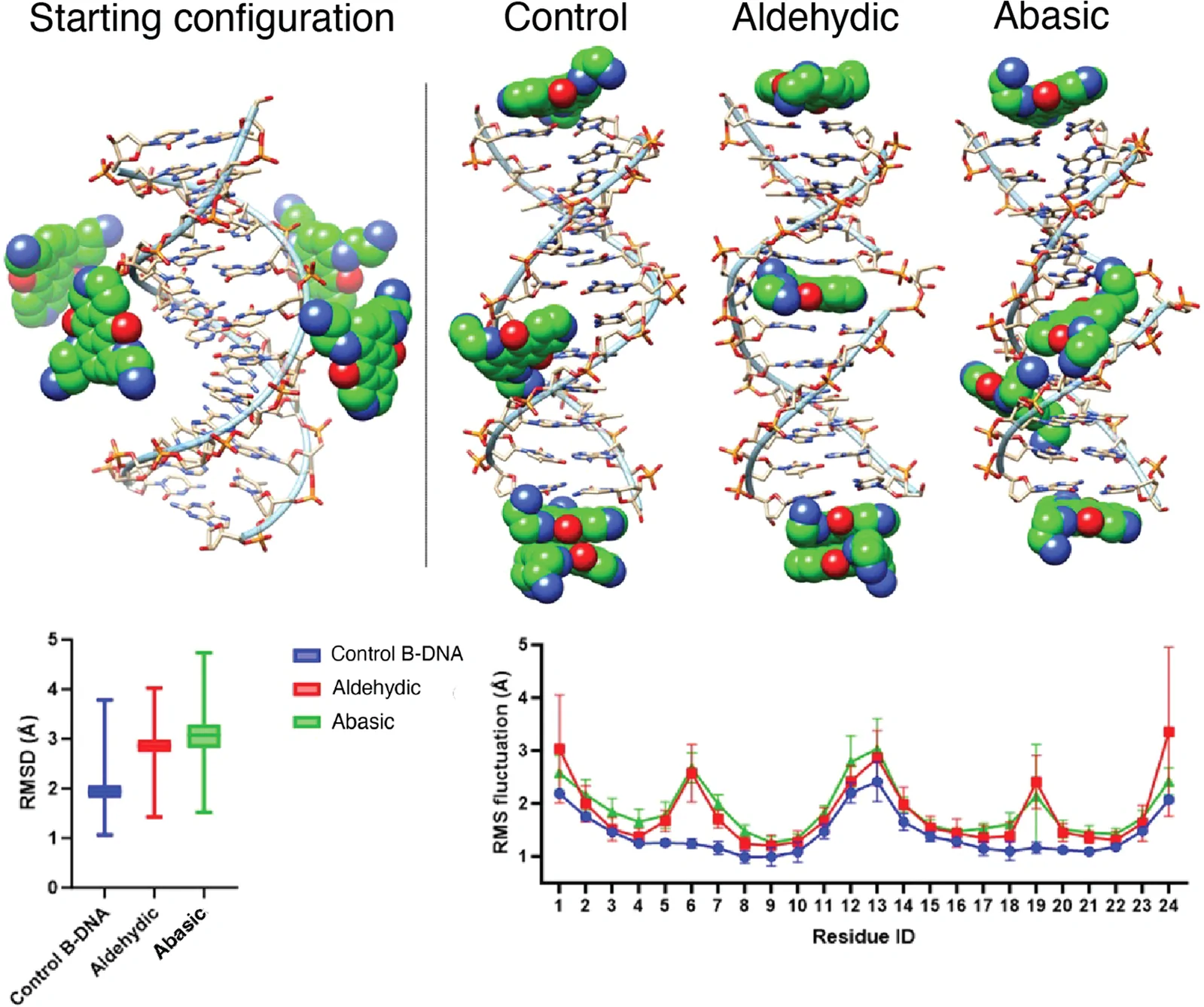
Top left -starting conformation of all the MD simulations. Top-right—representative structures of most populated clusters using five independent replicate. RMSD values calculated using the first frame as reference (showing SD). RMS fluctuations and RMSD values calculated using five independent replicate. Pixantrone carbon atoms are in green.

### NMR Analysis

The duplex 5′-d­(C^1^A^2^G^3^A^4^C^5^T^6^ X ^7^A^8^G^9^A^10^C^11^T^12^)-3′:5′-d­(A^13^G^14^T^15^C^16^T^17^C^18^A^19^G^20^T^21^C^22^T^23^G^24^)-3′ (X = reduced PIX-AP site conjugate), was analyzed by ^1^H NMR. Spectra were recorded over a temperature range of 278 to 308 K. This temperature range was below the *T*_m_ of the duplex, ensuring sharp NMR signals for the duplex DNA. The optimal temperature for collecting nonexchangeable ^1^H data was 288 K, while spectra of Watson–Crick hydrogen-bonded imino- and amino exchangeable protons were best collected at 274 K to minimize exchange broadening.

#### Nonexchangeable DNA Protons

The sequential ^1^H NMR assignments of the unmodified and the modified duplexes were completed using standard protocols (Figure 2).^62,63^ For the modified strand 5′-d­(C^1^A^2^G^3^A^4^C^5^T^6^ X ^7^A^8^G^9^A^10^C^11^T^12^) (X = reduced PIX-AP site conjugate) containing the reduced PIX-AP site conjugate, a continuous set of sequential NOEs of normal intensities was observed from C^1^ to T^6^. There was an interruption in the sequential NOEs between the T^6^ H6 and T^6^ H1′ protons. Two sets of sequential resonance signals were observed for nucleotides C^5^, T^6^ and A^8^. ^1^H COSY spectra afforded identification of ^3^ *J* COSY cross-peaks corresponding to vicinal couplings between the cytosine H5 and H6 protons in both the unmodified and modified duplexes. In agreement with the NOESY data, for the duplex containing the site-specific reduced pixantrone AP site conjugate, two COSY cross-peaks were observed for nucleotide C^18^ (Figure S1 in the Supporting Information). A continuous set of sequential NOEs of normal intensities was observed from and from A^8^ to T^12^. For the complementary strand 5′-d­(A^13^G^14^T^15^C^16^T^17^C^18^A^19^G^20^T^21^C^22^T^23^G^24^)-3′, a continuous set of sequential NOEs was observed (Figure 2). Two sets of signals were resolved from T^17^ to G^20^. The two NOEs between the T^17^ H1′ proton and the C^18^ H6 proton were of low intensity. The sequential NOEs from the C^18^ H1′ proton to the G^24^ H1′ proton were of normal intensities. The complete assignments of the deoxyribose H2′, H2″, and H3′ proton resonances were achieved. Most of the H4′ proton resonances were assigned. Partial assignments were made for the deoxyribose H5′ and H5″ proton resonances; these were in many instances overlapped precluding unequivocal assignments. The assignments of the DNA nonexchangeable protons are tabulated in Table S1 of the Supporting Information.

**Figure 2:**
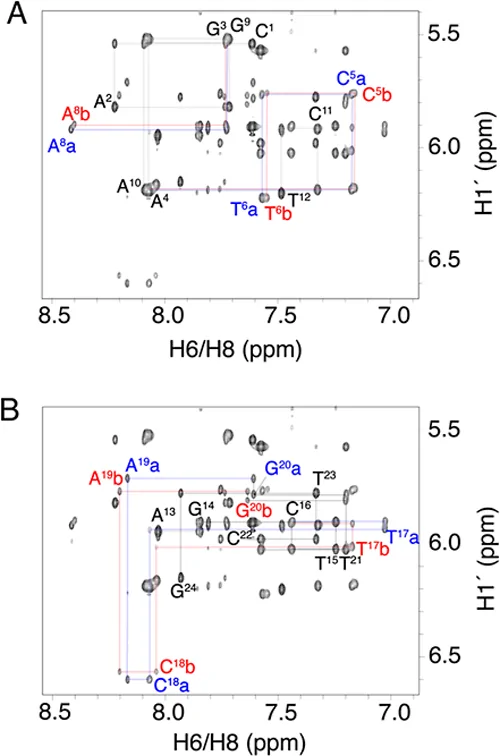
^1^H NOESY spectrum of 5′-d­(C^1^A^2^G^3^A^4^C^5^T^6^ X ^7^A^8^G^9^A^10^C^11^T^12^)-3′:5′-d­(A^13^G^14^T^15^C^16^T^17^C^18^A^19^G^20^T^21^C^22^T^23^G^24^)-3′ (X = reduced PIX-AP site conjugate) showing sequential NOEs between aromatic pyrimidine H6 or purine H8 protons and deoxyribosyl H1′ protons. (A) The modified strand, showing nucleotides C^1^ → T^12^. There is no aromatic proton on PIX showing an NOE with a deoxyribosyl proton in this region of the ^1^H spectrum. (B) The complementary strand, showing nucleotides A^13^ → G^24^. The spectrum was acquired at 900 MHz, at 250 ms mixing time. The temperature was 288 K.

#### Exchangeable DNA Protons

Figure 3 shows ^1^H spectra collected at 278 K in buffer containing 95:5 H_2_O/D_2_O, for the reduced PIX-AP site conjugate duplex 5′-d­(C^1^A^2^G^3^A^4^C^5^T^6^ X ^7^A^8^G^9^A^10^C^11^T^12^)-3′:5′-d­(A^13^G^14^T^15^C^16^T^17^C^18^A^19^G^20^T^21^C^22^T^23^G^24^)-3′ (X = reduced PIX-AP site conjugate). The guanosine N1H and thymine N3H imino proton resonances were sharp in ^1^H spectra collected at 278 K but broadened at higher temperatures. They were not observed at 298 K. The T^12^ N3H, T^15^ N3H, and T^17^ N3H imino resonances were partially resolved. The imino proton resonances were assigned using standard protocols;^64^ a ^1^H NOESY spectrum at 278 K is shown in Figure 3. The serial assignment T^23^ N3H → G^3^ N1H → T^21^ N3H → G^20^ N1H → T^6^ N3H, was obtained. The T^6^ N3H imino proton appeared as two distinct signals. The serial T^6^ N3H → C^18^ N3H → T^17^ N3H NOEs were not observed. The serial assignments T^17^ N3H → G^9^ N1H → T^15^ N3H → G^14^ N1H were obtained. The terminal imino proton resonances C^1^ N1H and T^12^ N3H were observed at δ 13.4 ppm and δ 12.9 ppm, respectively. They were broadened, presumably due to rapid exchange with water, in the NOESY spectrum. The T^23^ N3H resonance was assigned as the most downfield imino proton resonance (δ 14.1 ppm) because base pair A^2^/T^23^ is flanked by two G/C pairs, which enhance its stacking interactions. The remaining thymine imino protons, T^12^ N3H, T^15^ N3H, T^17^ N3H, and T^21^ N3H were within the anticipated δ 13.2–14.2 ppm range. The G^3^ N1H, G^9^ N1H, G^14^ N1H, G^20^ N1H, and G^24^ N1H imino proton resonances appeared in the anticipated δ 12.0–13.0 ppm range, characteristic of hydrogen-bonded G/C base pairs. G^14^ N1H (δ 12.4 ppm) was among the furthest upfield guanine resonances, indicating that the penultimate base pair C^11^/G^14^ had weaker base stacking, probably due to its positioning between base pairs A^10^/T^15^ and T^12^/A^13^, the latter being the terminal base pair. The chemical shift assignments for the exchangeable DNA protons are provided in Table S2 of the Supporting Information.

**Figure 3:**
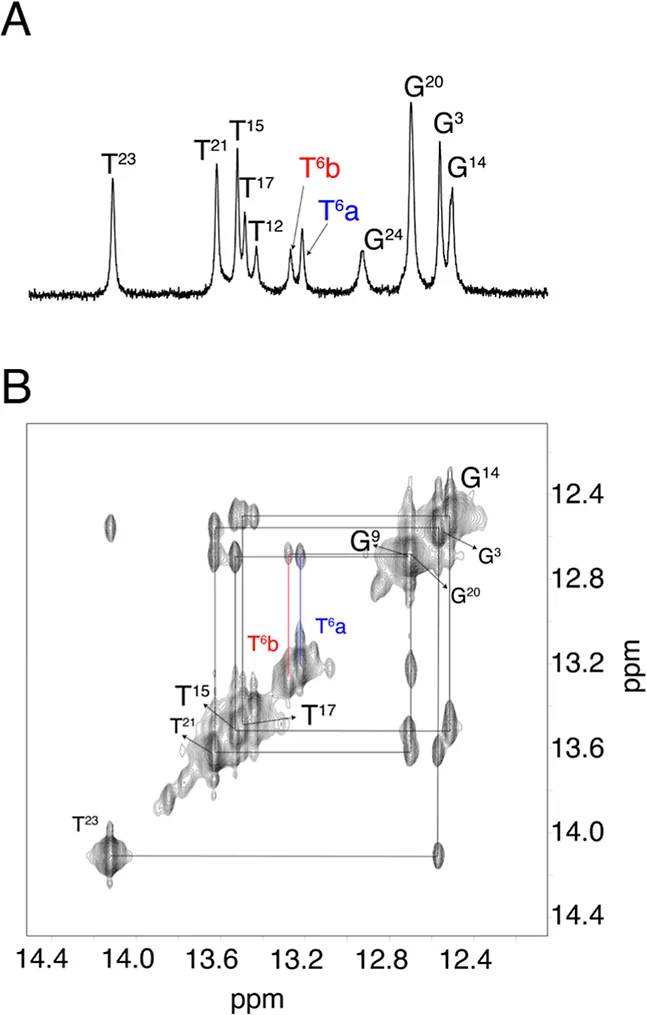
^1^H NMR of the modified duplex 5′-d­(C^1^A^2^G^3^A^4^C^5^T^6^ X ^7^A^8^G^9^A^10^C^11^T^12^)-3′:5′-d­(A^13^G^14^T^15^C^16^T^17^C^18^A^19^G^20^T^21^C^22^T^23^G^24^)-3′ (X = reduced PIX-AP site conjugate). (A) The thymine N3H and guanine N1H imino protons. (B) NOESY spectrum showing sequential NOE connectivities for the imino protons. The imino proton signals from regioisomer *R*_a_ are in blue, while the imino proton signals from *R*_b_ are in red.

#### PIX Protons

These reduced PIX-AP site conjugates form by nucleophilic attack by the terminal amines of the 2-aminoethyl side chains of pixantrone on the transient C1′ aldehyde associated with epimerization of AP sites, followed by reduction of the resulting Schiff bases (Scheme 1). The aza-anthracenedione ring renders the two 2-aminoethyl side chains nonequivalent (Chart 1). The NMR data confirmed the presence of two regioisomers, labeled as *R*_a_ and *R*_b_ (Figure 4). For each, the H3 and H4 protons were defined as the aromatic protons on the nitrogen-containing portion of the aza-anthracenedione exhibiting ^3^ *J* coupling; the H1 protons were defined as the aromatic protons on the nitrogen-containing portion of the aza-anthracenedione not exhibiting ^3^ *J* coupling. The two regioisomeric H3 signals appeared at δ 8.67 and δ 8.77 ppm, while the two regioisomeric H1 signals appeared at δ 8.64 and δ 8.74 ppm, due to their proximity to the nitrogen atom. The two regioisomeric H4 signals were observed further upfield. The pixantrone H7 and H8 protons were located on the carbon-containing ring, exhibited chemical shifts of δ 7.68 and δ 7.79 ppm, respectively, and were nearly isochronous. The spectral assignments of *R*_a_ and *R*_b_ regioisomers were made from assessments of NOEs to DNA protons (Figure 5). The regioisomer *R*_a_ was assigned by two unique and strong NOEs. The first was between pixantrone H4 and the T^17^ methyl protons in the complementary strand, the 3′ neighboring base pair with respect to PIX. The second was between pixantrone H1 and the T^6^ methyl protons, the 5′ neighboring nucleotide of PIX. These implied *R*_a_ was intercalated, bringing the pixantrone H1 proton proximate to the T^6^ methyl protons, and bringing pixantrone H4 proximate to the T^17^ methyl protons. For *R*_b_, a unique and strong NOE was observed between pixantrone H4 and the T^6^ methyl protons. This implied pixantrone H4 intercalated and was proximate to the T^6^ methyl protons. Both regioisomers displayed NOEs between the pixantrone H3 proton and the T^17^ methyl protons, but the intensity of this NOE was stronger for *R*_a_ than that for *R*_b_ (Figure 5). This was consistent with the chemical shifts of the regioisomeric pixantrone H3–H4 signals. In each, the more upfield signal was assigned to *R*_a_, as in this regioisomer, the H3 and H4 protons were positioned closer to the complementary cytosine, C^18^. In contrast, for *R*_b_, the H3 and H4 protons were oriented toward the major groove, resulting in a greater chemical shift compared to *R*_a_. The relative populations of each regioisomer were determined from integration. The integration of the signals arising from *R*_a_ were greater than the integrations of the signals assigned to *R*_b_. The ratio observed was 6:4 *R*_a_/*R*_b_.

**Scheme 1:**
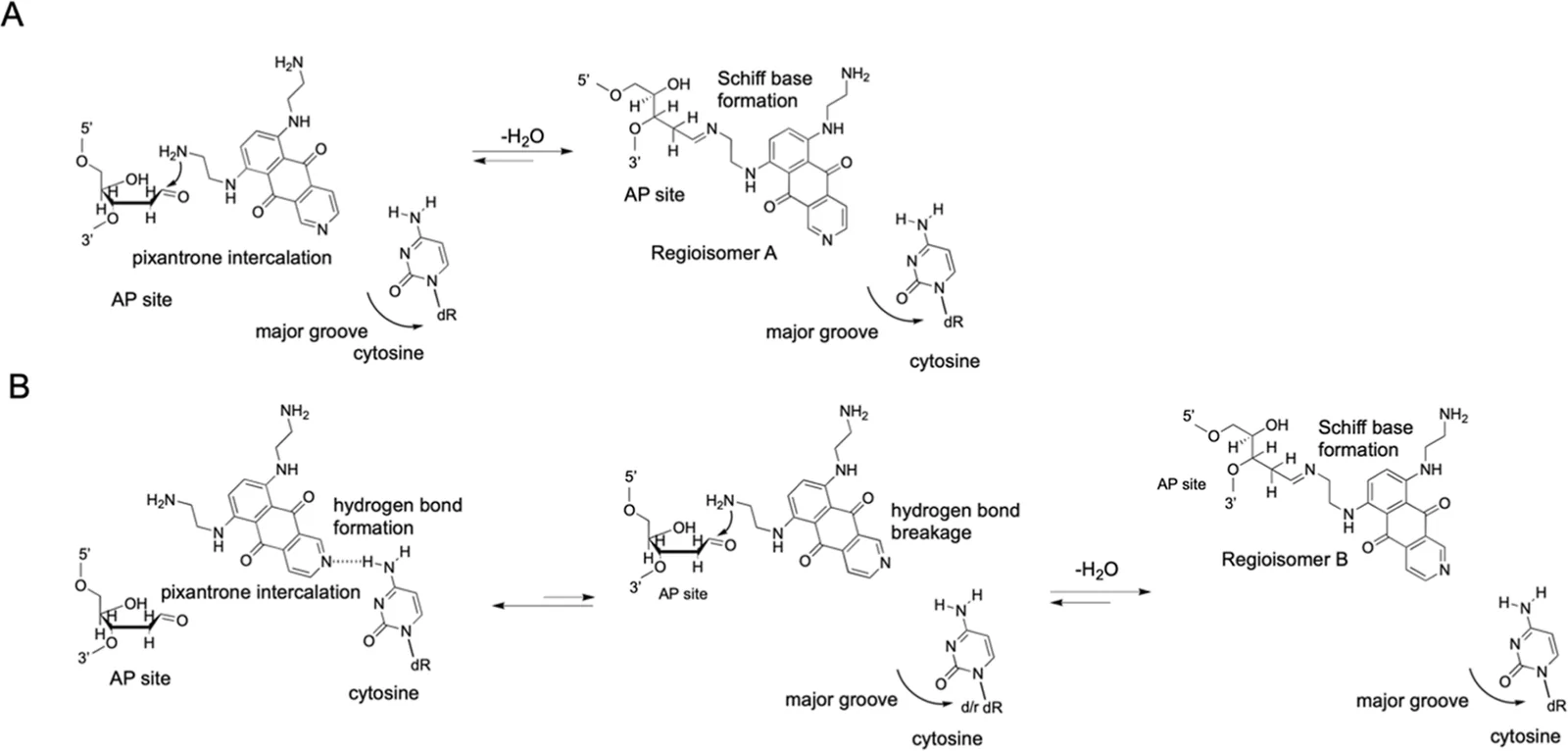
(A) Proposed Mechanism of Formation of *R*_a_; (B) Proposed Mechanism of Formation of *R*_b_

**Figure 4:**
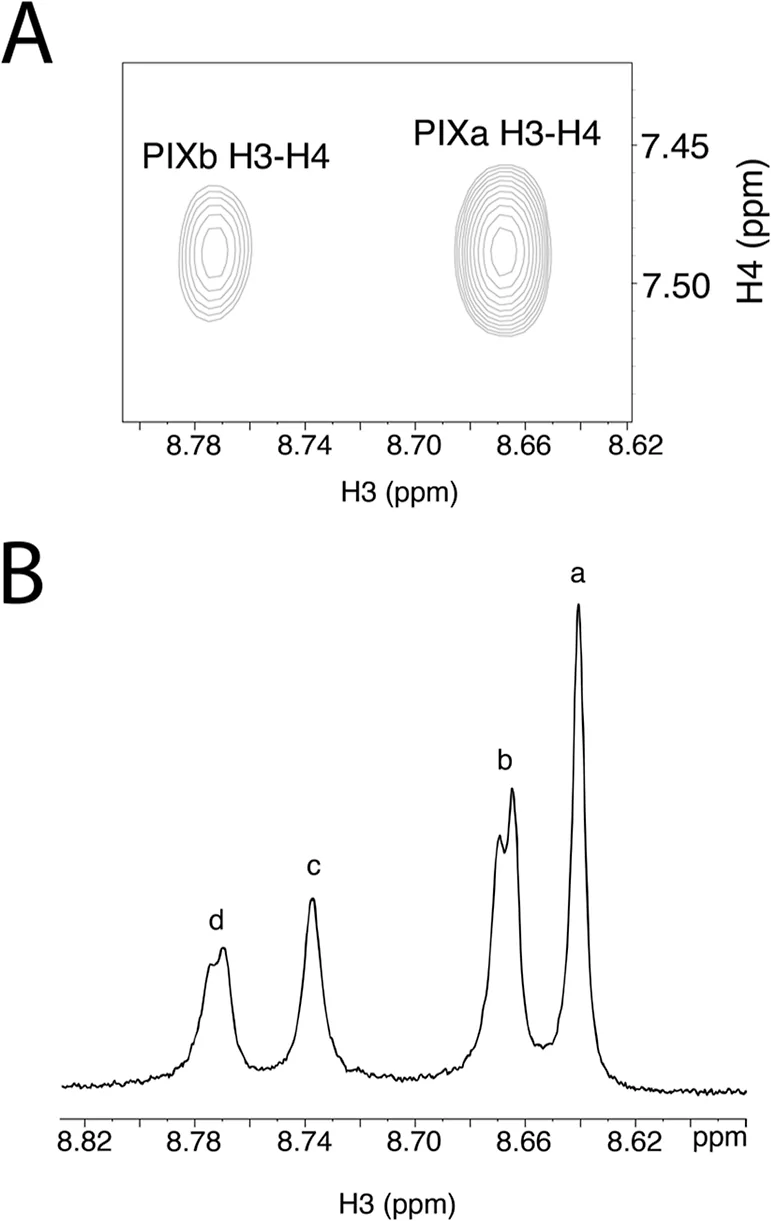
(A) ^1^H COSY spectrum of the modified duplex 5′-d­(C^1^A^2^G^3^A^4^C^5^T^6^ X ^7^A^8^G^9^A^10^C^11^T^12^)-3′:5′-d­(A^13^G^14^T^15^C^16^T^17^C^18^A^19^G^20^T^21^C^22^T^23^G^24^)-3′ (X = reduced PIX-AP site conjugate) showing scalar couplings between PIX H3 and H4 protons arising from the two regioisomers *R*_a_ and *R*_b_. The spectra were acquired at 900 MHz. The temperature was 288 K. (B) ^1^H NMR spectrum showing the PIX H1, H3 protons arising from the regioisomeric reduced PIX-AP site conjugates *R*_a_ and *R*_b_. The resonances are labeled a, *R*_a_ H1; b, *R*_a_ H3; c, *R*_b_ H1; d, *R*_b_ H3.

**Figure 5:**
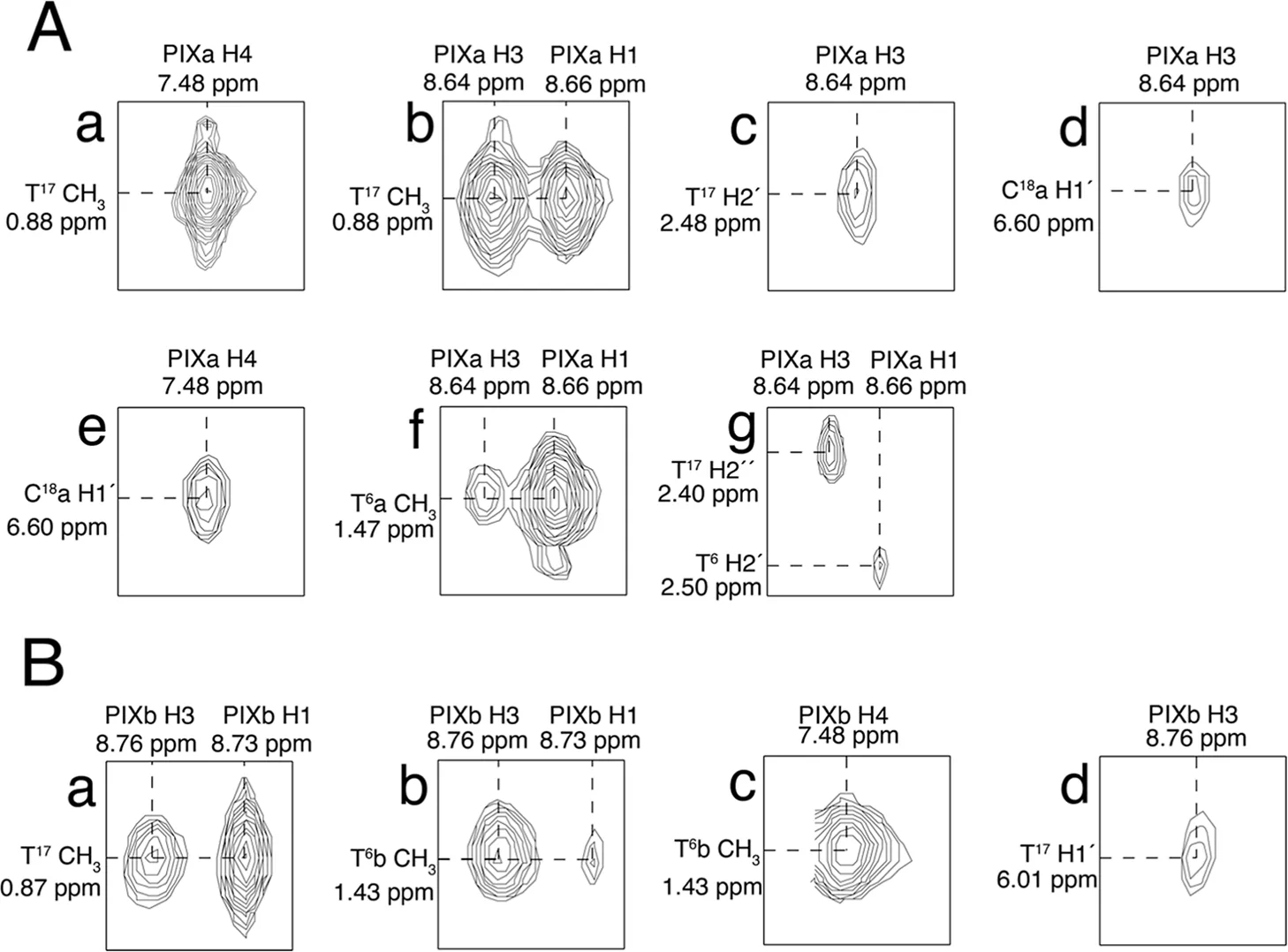
^1^H NOESY spectra of 5′-d­(C^1^A^2^G^3^A^4^C^5^T^6^ X ^7^A^8^G^9^A^10^C^11^T^12^)-3′:5′-d­(A^13^G^14^T^15^C^16^T^17^C^18^A^19^G^20^T^21^C^22^T^23^G^24^)-3′ (X = reduced PIX-AP site conjugate). (A) NOEs observed for regioisomer *R*_a_ with T^6^, T^17^, and C^18^ DNA protons. (B) NOEs observed for regioisomer *R*_b_ with T^6^, and T^17^ protons. a, *R*_b_ T^17^ CH_3_ → *R*_b_ X^7^ PIX H3; b, *R*_b_ T^17^ CH_3_ → *R*_b_ X^7^ PIX H1; c, *R*_b_ T^17^ CH_3_ → *R*_b_ PIX H6; d, *R*_b_ T^6^ CH_3_ → *R*_b_ X^7^ PIX H3; e, *R*_b_ T^6^ CH_3_ → *R*_b_ PIX H6; f, *R*_b_ T^6^ CH_3_ → *R*_b_ X^7^ PIX H4; g, *R*_b_ T^6^ CH_3_ → *R*_b_ C^5^ PIX H6; h, *R*_a_ T^17^ CH_3_ → *R*_a_ X^7^ PIX H3; I, *R*_a_ T^17^ CH_3_ → *R*_a_ X^7^ PIX H1; j, *R*_a_ T^17^ CH_3_ → *R*_a_ X^7^ PIX H4; k, *R*_a_ T^17^ CH_3_ → PIX H6; l, *R*_a_ T^6^ CH_3_ → *R*_a_ X^7^ PIX H1; m, *R*_a_ T^6^ CH_3_ → PIX H6; n, *R*_a_ T^6^ CH_3_ → *R*_a_ C^5^ H6; o, T^17^ CH_3_ → C^16^ H6.

### Chemical Shift Perturbations

Figure 6 shows DNA chemical shift perturbations induced in the 5′-d­(C^1^A^2^G^3^A^4^C^5^T^6^ X ^7^A^8^G^9^A^10^C^11^T^12^)-3′:5′ d­(A^13^G^14^T^15^C^16^T^17^C^18^A^19^G^20^T^21^C^22^T^23^G^24^)-3′ duplex, relative to the corresponding unmodified duplex. For both regioisomers *R*_a_ and *R*_b_, substantial changes occurred at the modified base pair X^7^/C^18^ and its immediate flanking 5′ and 3′neighbor base pairs. The greatest perturbations for both *R*_a_ and *R*_b_ were observed for the C^18^ nucleobase, which was complementary to the modified X^7^ nucleotide. In the unmodified duplex, the regioisomeric C^18^ H5–H6 COSY crosspeaks were observed at δ 5.68 and δ 7.40 ppm, respectively. For the reduced PIX-AP site conjugate duplex, the regioisomeric C^18^ H5 signals were observed at δ 6.17 and δ 6.21 ppm, and the two H6 signals were observed at δ 8.05 and δ 8.08 ppm. For the *R*_a_ regioisomer, C^18^ H6 shifted downfield by δ 0.68 ppm; for *R*_b_, C^18^ H6 shifted downfield by δ 0.55 ppm. The *R*_a_ C^18^ H5 proton also shifted downfield by δ 0.50 ppm and the *R*_b_ C^18^ H5 proton shifted downfield by δ 0.45 ppm. These chemical shifts are visible in Figure 5. There were also large downfield shifts observed for the anomeric protons of each regioisomer. For *R*_a_, C^18^ H1́ shifted downfield by δ 1.07 ppm; for *R*_b_, C^18^ H1́ shifted downfield by δ 1.18 ppm. Among the neighboring nucleotides displaying two subspectra due to the presence of the regioisomeric *R*_a_ and *R*_b_ conjugates, the 5′- neighboring nucleotide T^17^ H6 proton showed the greatest difference in chemical shift. The Δδ shift for T^17^ H6 was δ 0.40 ppm in the *R*_a_-modified duplex and δ 0.25 ppm in the *R*_b_-modified duplex. Chemical shift perturbations were also observed for the PIX protons. The PIX H3 and H4 protons were defined as the aromatic protons on the nitrogen-containing portion of the aza-anthracenedione exhibiting ^3^ *J* coupling; the H1 protons were defined as the aromatic protons on the nitrogen-containing portion of the aza-anthracenedione not exhibiting ^3^ *J* coupling.

**Figure 6:**
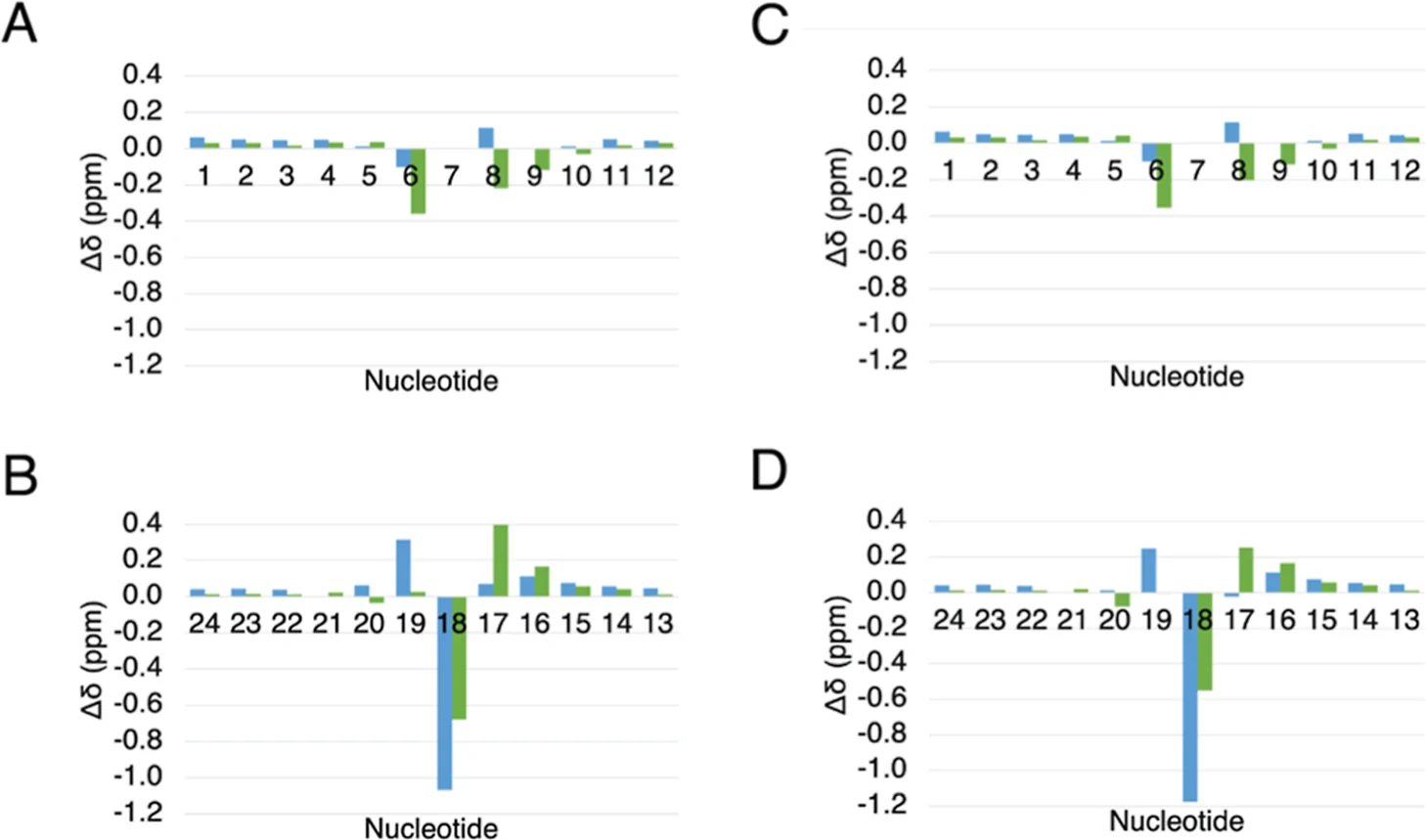
Chemical shift changes (Δδ) of pyrimidine H6 or purine H8 base protons (green) and H1́ protons (blue) of 5′-d­(C^1^A^2^G^3^A^4^C^5^T^6^ X ^7^A^8^G^9^A^10^C^11^T^12^)-3′:5′-d­(A^13^G^14^T^15^C^16^T^17^C^18^A^19^G^20^T^21^C^22^T^23^G^24^)-3′ (X = reduced PIX-AP site conjugate) relative to the corresponding unmodified duplex. (A) Template strand of the modified duplex containing regioisomer *R*_a_. (B) Complementary strands of the modified duplex containing regioisomer *R*_a_. (C) Template strands of the modified duplex containing regioisomer *R*_b_. (D) Complementary strand of the modified duplex containing regioisomer *R*_b_.

### Regioisomeric Reduced PIX-AP Site Conjugates

A series of reactions was conducted in 5′-d­(CAGACTXAGACT)-3′:5′-d­(AGTCTCAGTCTG)-3′ (X = reduced PIX-AP site conjugate) as a function of temperature (Figure 7). At 0 °C, the ratio of regioisomers as determined by NMR was *R*_a_/*R*_b_ = 1.8:1 after 30 min of incubation. No further changes were noted at longer incubation times. At 30 min of incubation at 35 °C, the ratio decreased to 1.5:1. At 35 °C, the ratio remained 1.5:1. At 60 °C, above the *T*_m_ for this duplex, the ratio decreased to 1.1:1. MALDI-TOF mass spectrometry confirmed the disappearance of residual AP site-containing duplex upon MX treatment (Figure S2 in the Supporting Information). Pixantrone was then reacted with the single strand 5′-d­(CAGACTXAGACT)-3′3′ containing an AP site. In these reactions, the *R*_a_/*R*_b_ ratio remained at 1:1, irrespective of incubation temperature (Figure 7). These data suggested that in dsDNA formation of regioisomer *R*_a_ was favored. A series of reactions were incubated at 0.5, 1, and 3 h, both at 0 and 35 °C. At 60 °C, complete reaction within 1 h was observed (Figure S3 in the Supporting Information). At both 0 and 35 °C, the ratio *R*_a_/*R*_b_ remained constant when the reactions were quenched at each time point (Figure S4 in the Supporting Information).

**Figure 7:**
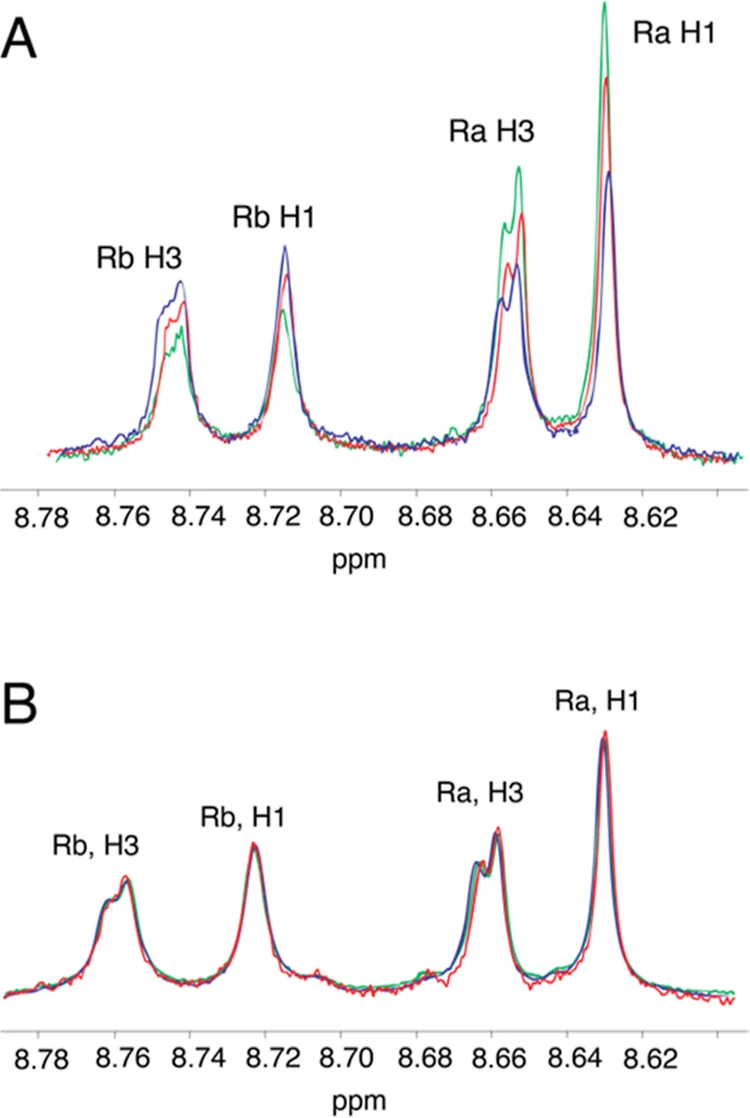
Overlay of ^1^H NMR spectra of the PIX H1 and H3 protons in 5′-d­(C^1^A^2^G^3^A^4^C^5^T^6^ X ^7^A^8^G^9^A^10^C^11^T^12^)-3′:5′-d­(A^13^G^14^T^15^C^16^T^17^C^18^A^19^G^20^T^21^C^22^T^23^G^24^)-3′ (X = reduced PIX-AP site conjugate) prepared either in dsDNA or ssDNA. (A) Synthesized in AP-containing dsDNA, incubated at 0 °C (green), 35 °C (red), and 60 °C (blue). (B) Synthesized in ssDNA, incubated at 0 °C (green), 35 °C (red), and 60 °C (blue). The reaction time was for 30 min.

### Unbiased MD Simulations of the Reduced PIX-AP Site Conjugates

To elucidate whether the position of the asymmetric isoquinoline N2 nitrogen of PIX would influence formation of the intercalated PIX-DNA system, we conducted simulations where PIX was covalently conjugated with the AP site. Two regioisomeric models were constructed of *R*_b_ or *R*_a_ (Figure 8). The intercalation process followed a similar mechanism for both regioisomers where PIX must reorient the aromatic component within the minor groove (Figure 9, panels B and F). As the simulations progressed, the bulge caused by the AP site allowed the unmatched cytosine base to freely rotate toward the solvent, exposing the gap between base-pairs C^8^/G^19^ and C^5^/G^21^ (Figure 9). Visual inspection of the MD trajectories suggested this step as the principal reorientation required to produce the final intercalated adduct. Exposing the gap caused by the flipping of C^20^ allowed PIX to insert in the resulting cavity, forming stacking interactions within the gap (Figure 9, panels D and H) and stabilizing with hydrogen bonds of the backbone at C^8^, G^19^ and G^21^ (Figure 10). All five independent simulations produced a similar PIX-DNA intercalated structure that included the unmatched cytosine flipped toward the major groove. No discernible difference was detected if the PIX asymmetric N2 atom started in either position 1 (*R*_b_) or position 2 (*R*_a_). Binding energy analysis showed energies for position 1 (*R*_b_) and position 2 (*R*_a_) of the intercalated structures of −102.2 (±8.3) and −108.1 (±9.7) kcal/mol, respectively. The event that was required to form the final intercalated structure was the flipping of the cytosine residue. Once it shifted conformation toward the major groove, the PIX readily inserted the isoquinoline ring within the cavity.

**Figure 8:**
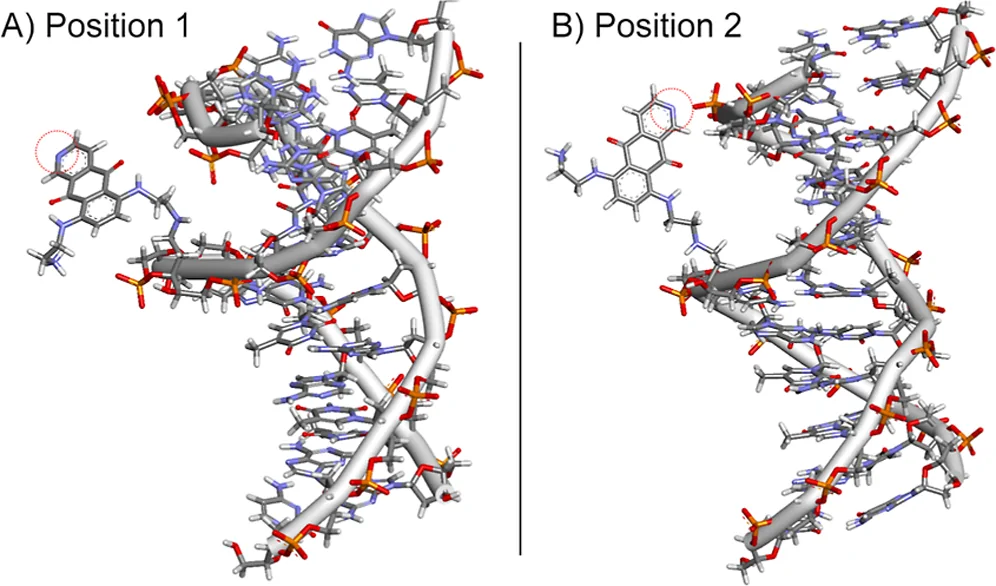
Starting positions of the reduced PIX-AP site conjugate with DNA for unbiased MD calculations. The regioisomeric difference is observed in the red circles showing the positions of the asymmetric N2 atoms .

**Figure 9:**
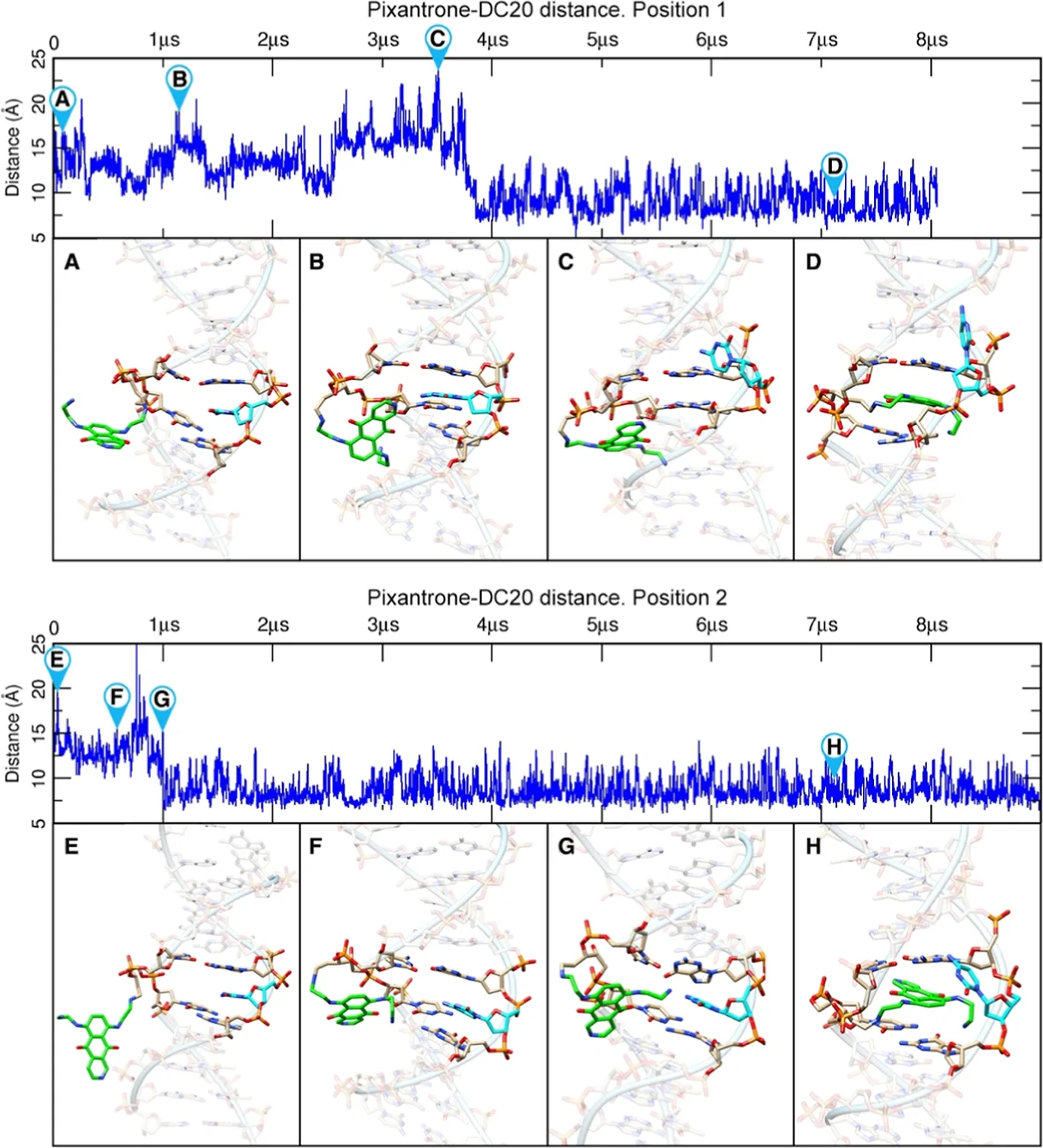
Unbiased MD calculation of the reduced PIX-AP site conjugate with DNA. Distance between the center of mass of PIX and cytosine 20. Panels A through H—selected frames from the trajectory to visualize the progress of the simulation. PIX is in green, unmatched cytosine 5 is in cyan.

**Figure 10:**
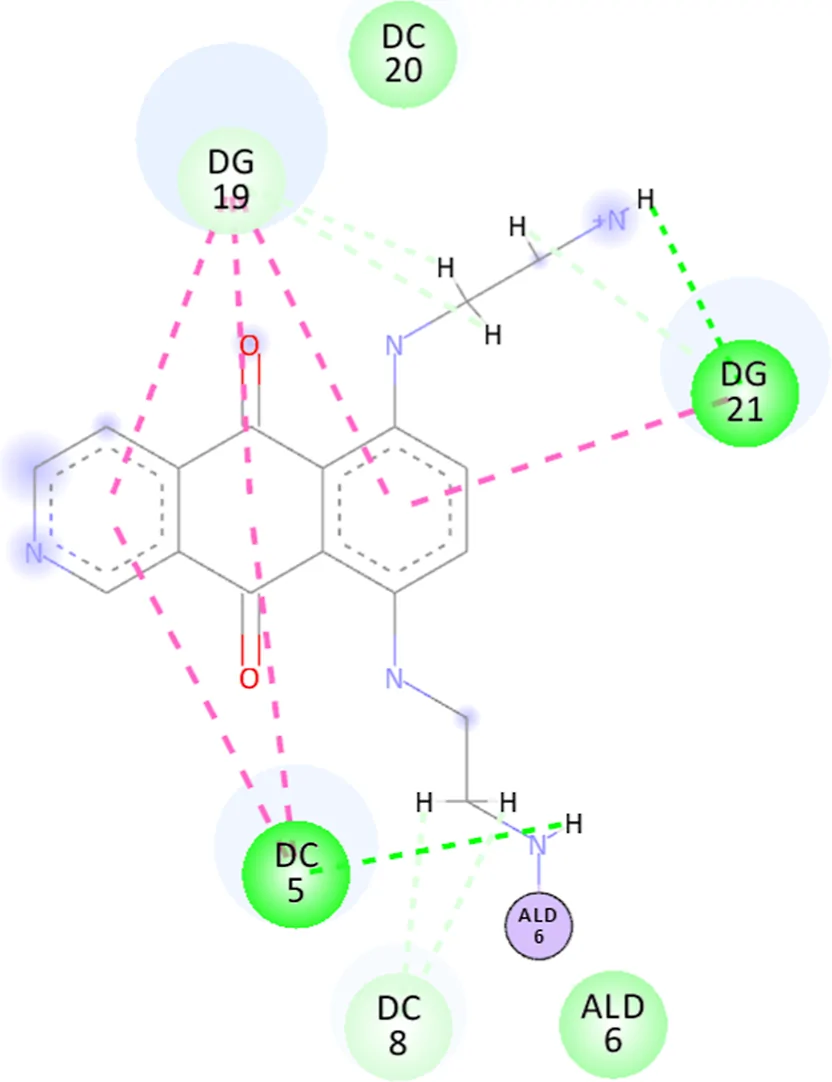
Specific interactions of the reduced PIX-AP site conjugate regioisomer *R*_a_ in the intercalated structure.

### Melting Temperature Analyses

Monophasic melting transitions were observed for both the 5′-d­(CAGACTXAGACT)-3′:5′-d­(AGTCTCAGTCTG)-3′ (X = reduced PIX-AP site conjugate, or THF) and as well, for the unmodified duplex (Figure 11). The *T*_m_ of the unmodified duplex 5′-d­(CAGACTGAGACT)-3′:5′-d­(AGTCTCAGTCTG)-3′was 58.8 °C. The THF containing duplex 5′-d­(CAGACTXAGACT)-3′:5′-d­(AGTCTCAGTCTG)-3′ (X = THF), which mimics an AP site, had a *T*_m_ of 37.2 °C. All the samples for melting temperature analyses were at 5 μM. Incorporation of the reduced PIX-AP site conjugate 5′-d­(CAGACTXAGACT)-3′:5′-d­(AGTCTCAGTCTG)-3′ (X = reduced PIX-AP site conjugate), stabilized the AP site relative to the THF-containing duplex; the *T*_m_ of the reduced PIX-AP site conjugate conjugate was 47.6 °C. This resulted in a 10.4 °C increase in the *T*_m_, but destabilized DNA compared to the unmodified form and resulted in a 11.2 °C reduction in the *T*_m_.

**Figure 11:**
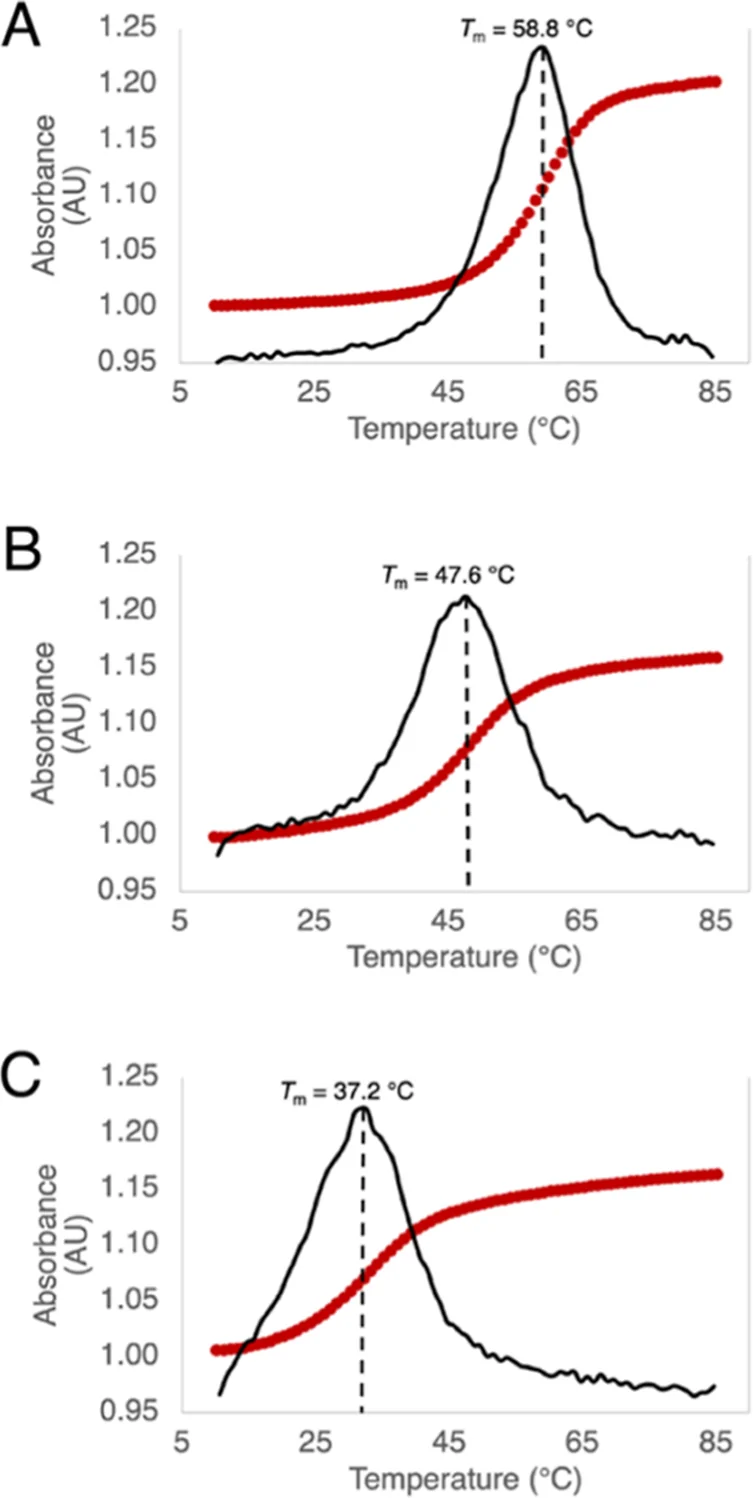
Thermal melting curves as recorded by absorbance at 260 nm. (A) The unmodified 5′-d­(CAGACTXAGACT)-3′:5′-d­(AGTCTCAGTCTG)-3′duplex. (B) The modified 5′-d­(CAGACTXAGACT)-3′:5′-d­(AGTCTCAGTCTG)-3′ (X = reduced PIX-AP site conjugate) duplex. (C) The modified 5′-d­(CAGACTXAGACT)-3′:5′-d­(AGTCTCAGTCTG)-3′ (X = THF) duplex. Melting curves are dotted-lines in red, first derivatives of melting curves are continuous-lines in black.

### Structural Refinement

#### NOEs Between Regioisomeric Pixantrone AP site Conjugates and DNA

For both regioisomers, nucleotides C^5^, T^6^, A^8^, and T^17^, C^18^, A^19^, G^20^ in the complementary strand close to the PIX-AP site conjugate showed subspectra arising from the two regioisomers (Figure 5). NOEs from those nucleotides were critical for differentiating the two subspectra defining the regioisomeric PIX-AP site conjugates. Strong intranucleotide NOE cross-peaks between H3 and H4 were observed for both regioisomers (Figure 4). For internucleotide NOEs, *R*_a_ exhibited interactions between PIX protons H1, H3, and H4 with protons on T^6^, T^17^, and C^18^ (Figure 5). In contrast, *R*_b_ showed NOEs between PIX protons H1, H3, and H4 with T^6^ and T^17^ but not with C^18^ (Figure 5).

#### Distance Restraints

The reduced PIX-AP site conjugates exist as a mixture of *R*_a_ and *R*_b_ regioisomers. To correct for this, intensities of NOEs observed for the *R*_a_ and *R*_b_ regioisomers were corrected by the ratio of regioisomers. The number of NOE-derived distance restraints from nonexchangeable protons calculated using MARDIGRAS^44^ was 189 for *R*_a_ and 185 for *R*_b_. These restraints were critical for defining structural differences between the two regioisomeric duplexes. For *R*_a_, 85 intranucleotide distance restraints, 91 internucleotide distance restraints, and 13 reduced PIX-AP-site conjugate specific restraints were identified. For *R*_b_, 85 intranucleotide, 89 internucleotide, and 11 reduced PIX-AP-site conjugate specific distance restraints were obtained. For both *R*_a_ and *R*_b_, 30 empirical Watson–Crick H-bonding restraints, 100 backbone torsion angle restraints, and 70 deoxyribose pseudorotation restraints were employed, based on the canonical B-DNA structure.^45^ Structural perturbations introduced by the reduced PIX-AP site conjugates affected base pairs T^6^/A^19^, X^7^/C^18^, and A^8^/T^17^. For both *R*_a_ and *R*_b_ canonical B-DNA restraints were not employed for nucleotides T^6^, X^7^, A^8^, T^17^, C^18^, and A^19^, or for the terminal nucleotides C^1^, T^12^, A^13^, and G^24^. In total, for *R*_a_ and *R*_b_, 394 and 390 experimental and canonical restraints were applied, respectively. The independent application of distance restraints for *R*_a_ and *R*_b_ ensured accurate structural determination of both conjugate duplexes. The complete list of restraints is provided in Tables S3 and S4 of the Supporting Information.

### Restrained Molecular Dynamics (rMD) Calculations

For structural refinement, initial models were constructed using MOE,^46^ followed by unrestrained potential energy minimization to relieve steric strain, particularly around the bulky PIX moieties. These were further processed in xLEAP,^47^ with partial charges calculated using the B3LYP/6-31G* basis set in GAUSSIAN.^25^ A series of 100 ps rMD calculations were performed independently for each regioisomer using a simulated annealing protocol.^48,50^ For both *R*_a_ and *R*_b_, experimental and canonical restraints were applied with a force constant of 30 kcal mol^–1^ Å^–2^ (Tables 1 and 2). Potential energy minimizations were performed using both steepest descent and conjugate gradient algorithms. The potential energy minimized structures were then used as the new starting structures, as pocket formation and unwinding were observed in the modification region. The coordinate and topology files were regenerated in xLEAP following the same protocol.^47^ Each of the 20 emergent structures for *R*_a_ and *R*_b_ underwent further potential energy minimization using the steepest descent algorithm followed by conjugate gradients. The rMD calculations for each regioisomer converged, producing 20 energy minimized structures that were used to generate an average structure via the program SUPPOSE.^48^

**Table 1:** NMR Restraints Used for the *R*_a_/*R*_b_-Modified Duplex Structural Refinement

	*R*_a_	*R*_b_
internucleotide restraints	122	117
intranucleotide restraints	109	99
PIX-AP restraints	11	9
total NOE restraints	231	216
backbone torsion angle restraints	85	85
hydrogen bonding restraints	35	35
deoxyribose pseudorotation restraints	80	80
total number of restraints	431	416
number of distance restraints violations	17	15
number of distance restraints violations	6	6
total distance penalty/maximum penalty (kcal/mol)	128.768/10.315	117.110/8.267
total torsion angle penalty/maximum penalty (kcal/mol)	2.0345/0.087	2.167/0.071
rms distances (Å)	0.0213	0.0189
rms angles (deg)	2.760	2.560
distance restraint force field (kcal/mol/Å^2^)	30	30
torsion angle restraint force field (kcal/mol/deg^2^)	30	30

**Table 2:** RMS Differences and Sixth Root Residual (*R* ^X^_1_) Values for the Average Structures of the *R*_a_/*R*_b_-Modified Duplex Emergent from rMD Calculations

	*R*_a_	*R*_b_
greatest RMS pairwise difference between 20 structures (Å)	1.25	1.02
greatest RMS difference from the average structures (Å)	1.06	0.097
sixth root residual *R* ^X^_1_ ^a^ calculated for the intranucleotide distances in the average structure, using CORMA^b^	0.0663	0.0703
sixth root residual *R* ^X^_1_ ^a^ calculated for the internucleotide distances in the average structure, using CORMA^b^	0.0675	0.0687
sixth root residual *R* ^X^_1_ ^a^ calculated for all distances in the average structure, using CORMA^b^	0.071	0.069
average error^c^	0.00089	0.00081

a *R* ^X^_1_ is the sixth root *R* factor Σ­[((*I*_o_)_i_^1/6^) – ((*I*_c_)_i_^1/6^Σ­((*I*_o_)_i_^1/6^].

b Mixing time was 250 ms.

c Average error: Σ­(*I*_c_ – *I*_*n*_)/*n*, where *I*_c_ is NOE intensities calculated from the refined structure, and *I*_*n*_ is experimental NOE intensities.

For *R*_a_, the average structure had a rms pairwise difference (rmsd) of 1.25 Å compared to the 20 individual structures. An overlay of these energy-minimized structures with the average minimized structure is shown in Figure 12. The structural analysis revealed that *R*_a_ intercalated into the duplex, with the complementary C^18^ nucleotide extruded into the major groove. For *R*_b_, the average structure had a pairwise rmsd of 1.02 Å, with its overlay also shown in Figure 12. For both regioisomers, base pair fraying was observed at the terminal regions at 288 K, leading to reduced convergence at the duplex ends.

**Figure 12:**
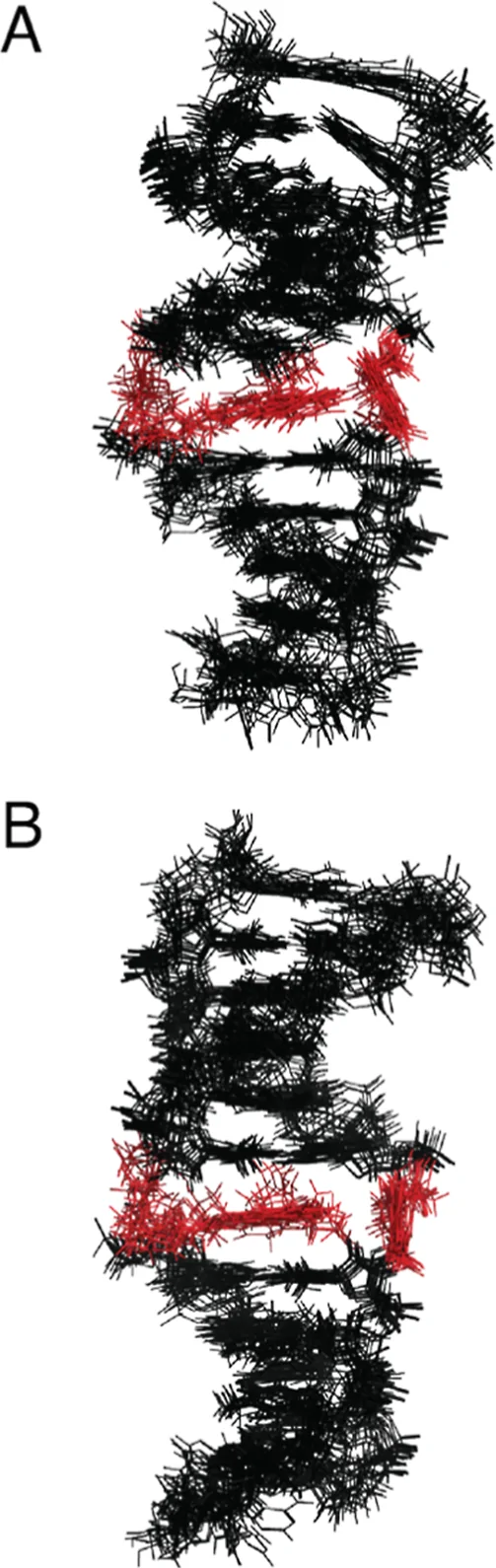
Overview of the 20 lowest energy violation structures generated from rMD calculations of (A) *R*_a_-modified duplex, and (B) *R*_b_-modified duplex, 5′- d­(CAGACTXAGACT)-3′:5′-d­(AGTCTCAGTCTG)-3′ (X = PIX-AP), carried out using a simulated annealing protocol and experimental NOEs generated distance restraints.

### Complete Relaxation Matrix Analysis

The accuracy of the average energy-minimized structures for *R*_a_ and *R*_b_ was evaluated using complete relaxation matrix analysis in CORMA.^44^ For *R*_a_, all intra- and internucleotide sixth root residuals *R* ^X^_1_ were equal to or less than 0.1, confirming an excellent agreement between the calculated and experimental NOE distances (Figure 13). The average *R* ^X^_1_ values for intranucleotide and internucleotide interactions were 0.0663 and 0.0675, respectively, indicating that the structures were in reasonable accordance with NOE data. For *R*_b_, a similar evaluation was performed. The individual intra- and internucleotide *R* ^X^_1_ values were also ≤0.1, suggesting excellent agreement with the experimental data (Figure 13). The average *R* ^X^_1_ values for intranucleotide and internucleotide interactions were 0.0703 and 0.0687, respectively. The overall *R* ^X^_1_ value for *R*_b_ was 0.0690. The complete relaxation matrix analysis confirmed that both regioisomeric structures were in reasonable agreement with the NOE-derived distance restraints. To assess consistency between model and experiment, CORMA analysis was performed. *R* ^X^_1_ values of all nucleotides were less than 0.1, indicating excellent agreement between calculated and experimental NOEs. To ensure that the observed difference was not an artifact of starting structure bias, simulated annealing calculations were repeated using cross-swapped minimized inputs (*R*_a_’s energy-minimized structure was used for *R*_b_’s protocol and vice versa). In both cases, the systems converged to similar final structures, with *R*_b_ consistently intercalated more deeply into DNA duplex than *R*_a_.

**Figure 13:**
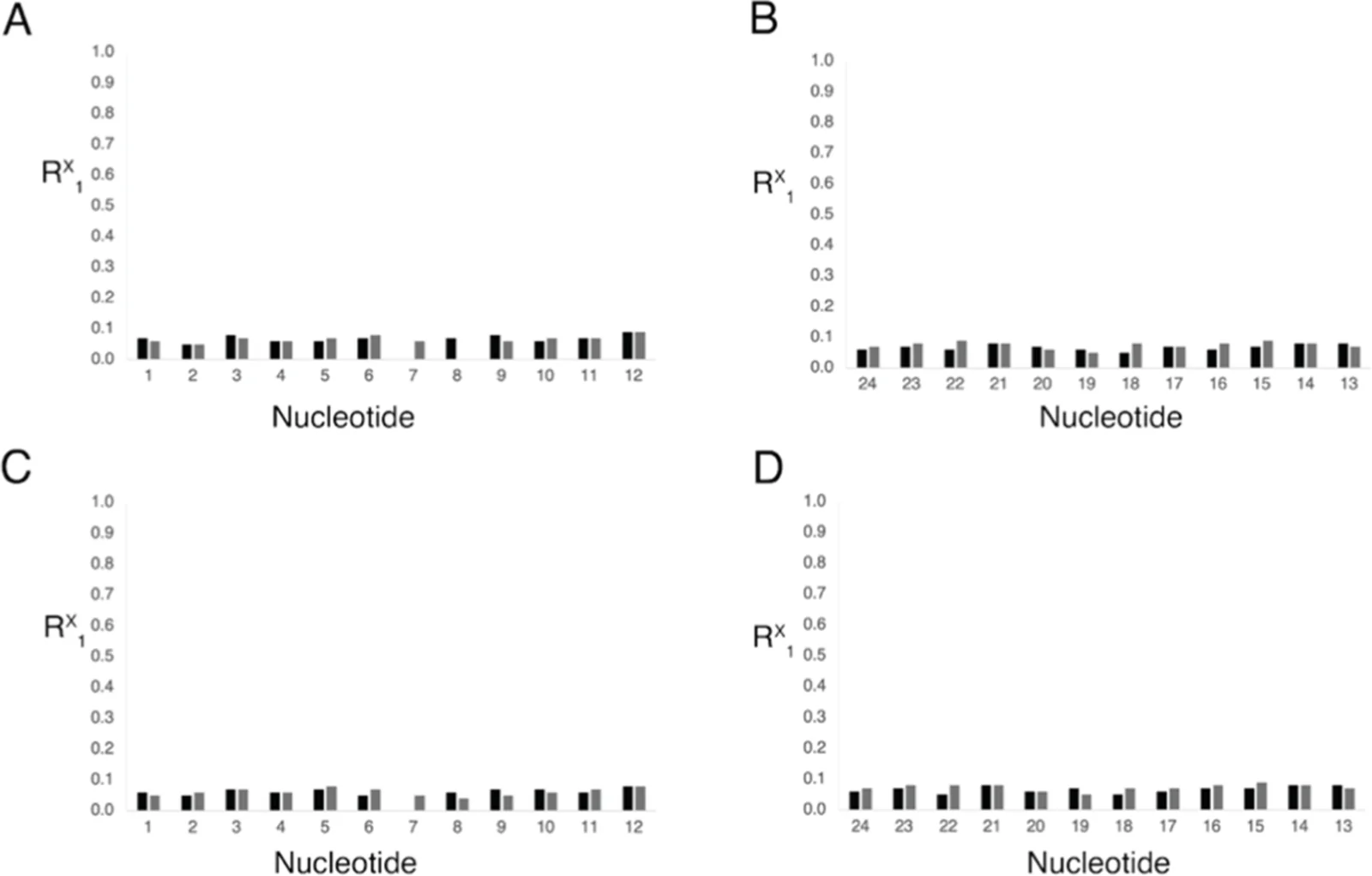
Calculation of sixth root residual values (*R* ^X^_1_) between theoretical NOEs predicted by complete relaxation matrix calculations and experimental NOEs for the averaged refined structure of 5′-d­(CAGACTXAGACT)-3′:5′-d­(AGTCTCAGTCTG)-3′ (X = PIX-AP) emergent from the rMD calculations, using CORMA. (A) The *R* ^X^_1_ values of intranucleotide and internucleotide NOEs in the *R*_a_-modified strand. (B) The *R* ^X^_1_ values of intranucleotide and internucleotide NOEs in the *R*_a_-modified complementary strand. (C) The *R* ^X^_1_ values of intranucleotide and internulceotide NOEs in the *R*_b_-modified strand. (D) The *R* ^X^_1_ values of internucleotide and intranulceotide NOEs in the *R*_b_-modified complementary strand. The intranucleotide NOE residuals are colored in black. The internucleotide NOE residuals are colored in gray.

### Structures of Regioisomeric Reduced PIX-AP Site Conjugate Duplexes

Figure 14 presents views of *R*_a_ and *R*_b_. Both intercalated into the DNA. For *R*_a_, the reduced PIX-AP site conjugate intercalated with the pixantrone N2 and H3 atoms facing into the major groove. The complementary C^18^ was extruded into the major groove, making space for the PIX-AP site conjugate. Structural perturbations were localized at base pair X^7^/C^18^ and its 5′- and 3′-flanking base pairs, T^6^/A^19^ and A^8^/T^17^. The flanking base pairs maintained Watson–Crick H-bonding. For *R*_b_, the PIX-AP conjugate also intercalated into the DNA, but with a key difference. The asymmetric PIX N2 atom was positioned toward the displaced C^18^ and was intercalated more deeply. Like in *R*_a_, C^18^ was also extruded from the duplex into the major groove, making space for the reduced PIX-AP site conjugate. The PIX N2 and H3 protons still faced toward the major groove, but the overall intercalation depth was deeper compared to *R*_a_ (Figure 14). Overall structural perturbations were similar, primarily affecting X^7^/C^18^, T^6^/A^19^, and A^8^/T^17^, while the flanking base pairs retained Watson–Crick H-bonding.

**Figure 14:**
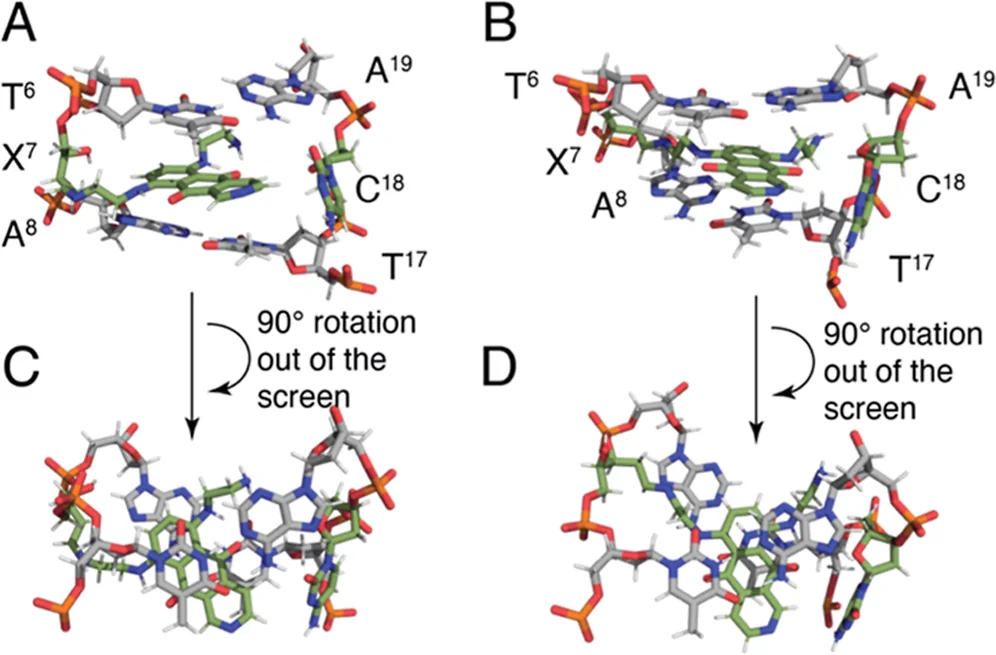
Base stacking of 5′-d­(CAGACTXAGACT)-3′:5′-d­(AGTCTCAGTCTG)-3′ (X = reduced PIX-AP site conjugate), showing base pairs T^6^/A^19^, X^7^/C^18^, and A^8^/T^17^, derived from the 20 lowest-energy structures from restrained MD calculations. The reduced PIX-AP site conjugates are green; other DNA bases are gray. (A) View from the major groove of the duplex containing regioisomer *R*_a_. (B) View from the major groove of the duplex containing regioisomer *R*_b_. (C) Top-down view of panel A rotated 90° outward from the screen. (D) Top-down view of panel B rotated 90° outward from the screen.

### Crystal Structure of a PIX-Modified DNA Dodecamer

The crystal structure of the modified DDD 5′-d­(C^1^G^2^C^3^X^4^A^5^A^6^T^7^T^8^C^9^G^10^C^11^G^12^)-3′ with a PIX-AP conjugate (X^4^) in place of G was determined by Br-SAD and refined to a resolution of 2.5 Å. X-ray crystallography revealed that replacing a guanine with the PIX-AP conjugate induces an abrupt structural kink, causing the DNA strands to form independent infinite duplexes connected by a perpendicular crossover duplex. Within these connector duplexes, the pixantrone (PIX) moieties do not intercalate into the DNA as traditionally expected; instead, they are flipped relative to one another in an extrahelical orientation and sandwiched between alternating C/G dimers to maximize stacking interactions. Selected crystal data, data collection parameters and final refinement parameters and deviations from ideal geometries are listed in Table S5 of the Supporting Information and the quality of the final electron density is depicted in Figure S5 of the Supporting Information. For a detailed description of the crystal structure see the Results and Discussion in the Supporting Information, and Figures S5–S8 in the Supporting Information.

## Discussion

Pixantrone,^65–67^ approved in the European Union for the treatment of relapsed or refractory aggressive B-cell non-Hodgkin lympohoma,^68,69^ was designed to reduce redox cycling associated with mitoxantrone, by eliminating the hydroquinone moiety.^70,71^ While PIX exhibits reduced cellular uptake as compared to MTX, it does not form stable Fe^3+^ complexes and shows minimal redox activity in cardiac tissue.^72^ The observation that PIX does not inhibit the topoisomerase IIb isoform found in cardiac tissues may also be a factor in its reduced cardiotoxicity as compared to MTX.^22^ More recently, Bellamri et al.^18^ demonstrated that amino groups of doxorubicin (DOX), MTX, and PIX may react with AP sites to form Schiff base conjugates. PIX differs from DOX or MTX since the presence of the asymmetric N2 nitrogen in PIX renders the (2-aminoethyl)­amino side chains nonequivalent. Consequently, unlike DOX or MTX, PIX has the potential to form regioisomeric PIX-AP site Schiff base conjugates.

### Regioisomeric PIX-AP Site Schiff Base Conjugates

Our results confirm that PIX forms regioisomeric Schiff base conjugates with AP sites in DNA, designated as regioisomers *R*_a_ and *R*_b_ (Figure 2). The reduced forms of these regioisomeric Schiff base conjugates are differentiated by their distinct patterns of ^1^H NOEs in NMR spectroscopy (Figure 5). Regioisomer *R*_a_ exhibits a unique strong NOE between PIX H4 and the T^17^ methyl protons in the complementary strand, the 3′ neighboring base pair with respect to PIX, as well as an NOE between PIX H1 and the T^6^ methyl protons, the 5′ neighboring nucleotide of PIX. The presence of these NOEs implies that for regioisomer *R*_a_ PIX is intercalated, bringing the PIX H1 proton proximate to the T^6^ methyl protons, and bringing PIX H4 proximate to the T^17^ methyl protons. In contrast, regioisomer *R*_b_ may be identified by a unique strong NOE between PIX H4 and the T^6^ methyl protons (Figure 5B). This implies that for regioisomer *R*_b_, PIX also intercalates, with PIX H4 proximate to the T^6^ methyl protons. Both regioisomers display NOEs between the PIX H3 proton and the T^17^ methyl protons, but with different intensities, that for *R*_a_ being stronger (Figure 5). This is consistent with chemical shifts of the regioisomeric PIX H3–H4 signals (Figure 4). For *R*_a_ PIX H3–H4 is the more upfield signal as in this regioisomer, the H3 and H4 protons are closer to the complementary cytosine, C^18^. In contrast, for *R*_b_, the PIX H3 and H4 protons orient toward the major groove, resulting in a greater chemical shift compared to *R*_a_.

### Regioisomeric Selectivity of PIX Intercalation into dsDNA

The 6:4 *R*_a_/*R*_b_ ratio of regioisomeric Schiff base conjugates in dsDNA observed by integration of NMR signals was unexpected. The PIX (2-aminoethyl)­amino side chains are distal to the asymmetric isoquinoline N2 nitrogen and might be anticipated to exhibit similar reactivity regarding forming Schiff base conjugates at AP sites. The differential formation of regioisomeric products does not appear to result from a thermodynamic preference for the reduced *R*_a_ vs *R*_b_ regioisomers in dsDNA. The single-phase melting curves observed for the regioisomeric mixture of reduced PIX-AP site conjugates (Figure 11) indicates that in dsDNA neither regioisomeric product is favored thermodynamically. As well, unbiased MD simulations show that both regioisomeric conjugates have similar intercalation energetics.

Instead, the distribution of regioisomeric products *R*_a_ and *R*_b_ in dsDNA may be determined by the relative rates of reaction leading to formation of each regioisomer, modulated by differences in intercalative binding of PIX to DNA. The asymmetric structure of PIX allows two orientations for intercalation of the isoquinoline ring at an AP site in dsDNA. In forming the *R*_a_ regioisomer, upon intercalation into dsDNA, the PIX asymmetric nitrogen N2 faces outward from the DNA helix. Intercalation of PIX in this orientation positions its side chain amino group in alignment with the AP site (Scheme 1). Once intercalated, the PIX amino group may attack the transiently formed aldehyde at the AP site, forming a Schiff base conjugate. In contrast, in forming the *R*_b_ regioisomer, upon intercalation, the PIX asymmetric nitrogen N2 orients toward cytosine C^18^ opposite the AP site. It comes within H-bonding distance of C^18^. When intercalated in this manner, an additional reorientation may be required before the PIX amino group can attack the AP site, forming the Schiff base conjugate. This may hinder formation of *R*_b_ in dsDNA. Significantly, in ssDNA containing an AP site, where the complementary cytosine C^18^ is absent, both regioisomers *R*_a_ and *R*_b_ form in equal proportions. Also, in dsDNA regioisomer *R*_a_ remains the predominant product if the reaction occurs below the *T*_m_ value of the dsDNA. Above the *T*_m_ value, regioisomer formation reverts to 1:1 *R*_a_/*R*_b_. Overall, regioisomer formation in dsDNA appears to be governed by the ability of the PIX asymmetric nitrogen atom N2 to interact with the complementary cytosine C^18^. This represents one possible interpretation based on NMR-derived structural evidence and should be viewed as a plausible explanation rather than a definitive mechanistic conclusion.

### Conformations of Reduced Regioisomeric Pixantrone-AP Site Conjugates

In both regioisomers the PIX isoquinoline ring stacks between neighboring base pairs, with the complementary C^18^ extruded into the major groove (Figure 14). The core dsDNA conformation is locally disrupted at the conjugated AP site and its flanking 5′ and 3′ neighboring base pairs, as evidenced by the observation that only C^5^, T^6^, A^8^, T^17^, C^18^, A^19^, and G^20^ exhibit two subspectra arising from the two regioisomers (Figures 2 and 3). Of the regioisomeric AP site conjugates *R*_b_ intercalates more deeply than *R*_a_. Overall, both regioisomeric PIX-AP site conjugates thermally stabilize the AP-containing dsDNA duplex (Figure 11). While both regioisomeric conjugates display NOEs between PIX H3 and the T^17^ methyl protons (Figure 5), the PIX H3 proton is closer to the T^17^ methyl group in *R*_a_ vs *R*_b_ (Figure 14) and the corresponding magnitude of the NOE is greater (Figure 5). The positioning of the asymmetric isoquinoline ring nitrogen N2 governs intercalation depth of the *R*_a_ and *R*_b_ regioisomeric AP site conjugates. The deeper intercalation of *R*_b_ may result due to the propensity of the N2 nitrogen to transiently hydrogen bond with the exocyclic *N* ^4^-dC amino group, thus pulling the PIX ring deeper into the duplex DNA. Thus, the regioisomeric differences in PIX N2 positioning may govern formation of regioisomeric PIX-AP conjugates.

### Anthracycline Antibiotics and Schiff Base Formation

AP sites in DNA are reactive electrophilic sites that may form covalent adducts with nucleophilic groups via a transiently formed aldehyde. The amino groups in anthracycline antibiotics are known to form covalent aminal linkages with DNA. In the presence of formaldehyde, the daunosamine N3′ amine of DOX conjugates via an aminal linkage with the exocyclic amino group of guanine in dsDNA; this chemistry facilitated crystallographic analyses of intercalated DNA-DOX adducts.^73–75^ These conjugates interfered with transcription factors that recognize 5′-GpC-3′ sequences, ultimately triggering apoptosis.^76^ PIX also forms aminal conjugates with the exocyclic amine of guanine in dsDNA in the presence of formaldehyde, in vitro.^77^ This leads to the formation of interstrand cross-links (ICLs) (Scheme 1).^12,78–86^ AP sites may generate stable interstrand cross-links (ICLs) with dA in dsDNA. Kellum et al.^13^ characterized a β-*N*-glycosyl linkage between an *N* ^6^-dA amino group and an AP site.

### Biological Implications

Anthracycline antibiotics are extensively used to treat various cancers due to their ability to disrupt cell division.^87,88^ Their primary mechanism of action is believed to be mediated by topoisomerase II inhibition,^89,90^ which leads to the induction of double-strand breaks, interference with DNA replication, and ultimately, cell death. Utilization of DOX and MTX in the clinic is limited by dose-dependent cardiotoxicity.^91,92^ The source is not completely understood and possibly involves multiple factors. It has been linked to quinone–hydroquinone redox cycling,^93^ and off-target collateral damage to the topoisomerase IIb isoform found in cardiac tissues.^22^ Both DOX and MTX form Fe^3+^ complexes and generate hydroxyl radicals,^94–96^ which promote iron-mediated oxidative stress.

The observation that PIX conjugates to AP sites in DNA as a mixture of regioisomeric products leads to the question as to whether *R*_a_ and *R*_b_ may be differentially recognized by DNA damage repair processes. Both regioisomeric reduced Schiff base conjugates are less thermodynamically stable compared to unmodified DNA, as shown by *T*_m_ studies (Figure 11), suggesting that PIX intercalation stabilizes the AP site but that the resulting Schiff base conjugate does not restore native dsDNA stability. This is consistent with reports that the Schiff base conjugates are reversible and have short lifetimes in dsDNA. Minko et al.^97^ reported PIX exhibited weaker AP lyase activity and showed less inhibition of APE1 as compared to MTX, attributed to its lower binding affinity for AP site-containing DNA. The reduced Schiff base regioisomer *R*_b_ inserts more deeply into the dsDNA helix than does the regioisomer *R*_a_. By comparison, the unique crystal structure observed for the DDD with PIX conjugated to AP sites at the G-tract/A-tract boundaries clearly supports the paramount role of stacking by the pixantrone anthracycline analog in organizing these conjugates.

### Summary

PIX forms regioisomeric Schiff base conjugates with AP sites in dsDNA, differing in the position of the asymmetric isoquinoline nitrogen N2. *R*_a_ predominates in dsDNA due to a favorable intercalation alignment, while *R*_b_ forms more slowly, possibly due to the need to break a stabilizing hydrogen bond with the opposing cytosine. In ssDNA, both regioisomers form in equal amounts. Our results suggest that formation of PIX regioisomeric Schiff base conjugates is sensitive to DNA environment, such as the presence of unwound replication forks, or supercoiled regions, which may modulate formation of the two regioisomers.

## Supplementary Material



## Notes

Supporting results and discussion for X-ray crystallographic studies, Tables S1, chemical shifts of nonexchangeable DNA protons; S2, chemical shifts (ppm) of exchangeable DNA protons; S3, NOE distance restraints of PIX-AP conjugate (*R*_a_) DNA duplex used for rMD calculations in AMBER; S4, NOE distance restraints of PIX-AP conjugate (*R*_b_) DNA duplex used for rMD calculations in AMBER; S5, selected crystal data, X-ray data collection and refinement parameters; and Figures S1, two-dimensional COSY spectra of PIX-AP conjugate duplex and unmodified duplex; S2, MALDI-TOF mass spectrum of the MX quenched reaction; S3, HPLC-UV chromatogram of the reaction of the AP-containing oligonucleotide with PIX reduced with NaB­(CN)­H_3_ at 60 °C analyzed after 1 h; S4, HPLC-UV chromatogram of the reaction of the AP-containing oligonucleotide with PIX reduced with NaB­(CN)­H_3_; S5, final Fourier 2F_o_-F_c_ sum electron density (green meshwork) around the C^3^–PIX-A^5^ base steps drawn at ∼1.1 σ threshold; S6, lattice interactions in the crystal structure of a modified DDD with a PIX-AP conjugate; S7, superimposition of yellow and green DDD strands with PIX-AP conjugates; S8, stacked PIX moieties alternating with CG dimers in the connector duplex (PDF)
