# Supplementary material for: Characterization of Regioisomeric Conjugates Arising from Pixantrone at AP Sites in DNA
Source: Biochemistry. 2026 Sep 4;65(19):3026–43. doi: 10.1021/acs.biochem.6c00116 (PMC13648238; doi:10.1021/acs.biochem.6c00116)
Supplement: Supplementary file 1 [file bi6c00116_si_001.pdf]

Characterization of Regioisomeric Conjugates Arising from Pixantrone at AP Sites in DNA

Yizhi Fu[1], John T. Terrell[1], Andrew H. Kellum Jr.[1], Pradeep S. Pallan[2], Rodrigo Galindo-Murillo[3], Carmelo J. Rizzo[1], Martin Egli[2,4,5] and Michael P. Stone[1,4,5,*]

Supporting Information

[1]Department of Chemistry, Vanderbilt University, Nashville, TN 37235, USA

[2]Department of Biochemistry, Vanderbilt University, School of Medicine, Nashville, TN 37232, USA

[3]Ionis Pharmaceuticals Inc., Carlsbad, CA 92010, USA

[4]Center for Structural Biology, Vanderbilt University, Nashville, TN 37235, USA,

[5]Vanderbilt-Ingram Cancer Center, Vanderbilt University Medical Center, Nashville, TN 37232, USA

*To whom correspondence should be addressed:

Michael P. Stone

615-322-2589

michael.p.stone@vanderbilt.edu

Table of Contents

1. **Supporting Results and Discussion for X-ray Crystallographic Studies Page 3**

2. **Supporting Tables**

S1. Chemical shifts (ppm) of non-exchangeable DNA protons. Page S5

S2. Chemical shifts (ppm) of exchangeable DNA protons. Page S7

S3. NOE distance restraints of PIXa-AP conjugate (R_a) DNA duplex used for rMD calculations in AMBER. Page S8

S4. NOE distance restraints of PIXb-AP conjugate (R_b) DNA duplex used for rMD calculations in AMBER. Page S15

S5. Selected crystal data, X-ray data collection and refinement parameters. Page S22

3. **Supporting Figures**

S1. Two-dimensional COSY spectra of PIX-AP conjugate duplex and unmodified duplex. Page S24

S2. MALDI-TOF mass spectrum of the MX quenched reaction. Page S25

S3. HPLC-UV chromatogram of the reaction of the AP-containing oligonucleotide with PIX reduced with $NaB(CN)H_3$ at 60 °C analyzed after 1 h. Page S26

S4. HPLC-UV chromatogram of the reaction of the AP-containing oligonucleotide with PIX reduced with $NaB(CN)H_3$. Page S27

S5. Final Fourier $2F_o$-F_c sum electron density (green meshwork) around the C^3-PIX-A^5 base steps drawn at ~1.1 σ threshold. Page S28

S6. Lattice interactions in the crystal structure of a modified DDD with a PIX-AP conjugate. Page S29

S7. Superimposition of yellow and green DDD strands with PIX-AP conjugates. Page S31

S8. Stacked PIX moieties alternating with CG dimers in the connector duplex. Page S32

Results and Discussion

X-ray Crystallography. The crystal structure of the modified Dickerson-Drew Dodecamer (DDD) 5′ -d($C^1G^2C^3X^4A^5A^6T^7T^8C^9G^{10}C^{11}G^{12}$)-3′ with a PIX-AP conjugate (X^4) in place of G was determined by Br-SAD and refined to a resolution of 2.5 Å (**Figure S5**). The structure in the hexagonal space group $P6_422$ revealed two independent 12-mer strands, four ethylene glycol molecules and 25 waters. Residues in both strands are numbered 1 to 12 and are colored in yellow (strand A) and green (strand B) in **Figure S6**. Nucleotides A^5 to G^{12} of both strands were part of an infinite duplex with A's and T's pairing in the core and extended by symmetry-related 12-mers that used their $C^9G^{10}C^{11}G^{12}$ ends to pair with the $C^9G^{10}C^{11}G^{12}$ ends of the parent strands under formation of C:G stretches that flanked the central A:T pairs (**Figure S6 A,B**). Between A^5 and the PIX-AP conjugate, both yellow and green strands underwent a sharp turn and looped away from the duplex formed by shifted and overlapping $A^5A^6T^7T^8C^9G^{10}C^{11}G^{12}$ octamers. The abrupt change in strand direction left the central dodecamer duplex virtually straight but resulted in pronounced kinks into the major groove at the transitions between G-tracts and A-tracts at both ends (see the overlaps with #2y and #4g symmetry mates depicted in **Figure S6 A**). The Pix conjugate along with $C^1G^2C^3$ of both the yellow and green strands and symmetry-related $C^1G^2C^3X^4$ tetramers then formed another duplex that crossed the first one at a roughly 90° angle and connected infinite duplexes assembled from yellow and green octamer strands. As schematically shown in **Figure S6 B**, looped out green and yellow $C^1G^2C^3X^4$ strand portions were arranged in a consecutive fashion such that the green tetramer continued the stack initiated by the yellow tetramer, with complementary pieces contributed by symmetry-related strands (#1g and #1y). Symmetry mates #3g, #3y, #5y and #5g then completed the crossover to the other infinite duplex. In this fashion, the connectors between infinite duplexes were composed of alternating C:G dimer steps and stacked PIX moieties sandwiched between them (**Figure S6**).

An overlay of yellow and green dodecamers revealed that their $A^5A^6T^7T^8C^9G^{10}C^{11}G^{12}$ octamer portions adopted virtually identical conformations (**Figure S7**). With one exception residues adopted Southern (C2′-endo, C3′-exo) or Southeastern (C1′-exo) deoxyribose puckers. C^{11} of the yellow strand had an Eastern pucker (O4′-endo). Yellow and green strands kinked in different directions and the latter assumed a more extended conformation that allowed it to stack onto the 5′ -end (G^2) of the yellow strand with its pixantrone moiety (**Figure S6 A**).

In the connector duplexes, stacked PIX moieties were twisted by ca. 36° as they alternated with stacked C:G dimers (**Figure S8**). At the current resolution of the structure, it was not possible to determine the orientation of the aza-anthracene-9,10-dione nitrogen. Thus, the position of that nitrogen

in the PIX moiety colored in green roughly below O2 of the cytosine colored in pink in **Figure S8** is arbitrary. As well, the position of the aza-anthracene-9,10-dione nitrogen in the PIX moiety colored in pink roughly above N4 of the cytosine colored in green in **Figure S8** is arbitrary. These two PIX moieties are not symmetry-related but belong to a green DDD strand and a symmetry mate of a yellow DDD strand (pink PIX). Neither the outer ring of the PIX moiety from one DDD nor that of the pairing DDD is positioned in the vicinity of an H-bond donor or acceptor. The absence of a direct or water-mediated interaction between DNA and PIX ring did not allow us to firmly establish which one of the two atoms forming the outer bond is nitrogen and which one is carbon.

As is evident from **Figures S7 B** and **S8**, symmetry-related yellow and green DDD strand portions that established a rung between infinite duplexes were 5′ - 3′ end-to-end stacked such that CG dimers formed discontinuous (interrupted by stacked PIX moieties) major and minor grooves. In other words, O6 of G and N4 of C were directed into the pseudo major groove and N2 of G and O2 of C were directed into the pseudo minor groove along the entire stretch. Conversely, conjugated stacked pixantrones were flipped relative to one another, such that the aminoethyl moiety of one (green) sat in the minor groove and that of the other (pink) sat in the major groove (**Figure S8**). The extrahelical orientation of pixantrone in the crystal structure and the observed arrangement of two pixantrones sandwiched by C:G pairs that served to maximize stacking strongly suggested that PIX-AP conjugate formation did not require the drug to be intercalated into DNA.

Table S1. Chemical shifts (ppm) of non-exchangeable DNA protons. N.A. = not applicable; n.o. = not observed; o. = overlapped.

Nucleotide	H6/H8	H5/CH_3	H1'	H2'	H2''	H3'
C^1	7.62	5.92	5.56	1.80	2.28	4.66
A^2	8.23	N.A.	5.84	2.75	2.83	5.01
G^3	7.72	N.A.	5.54	2.60	2.70	4.39
A^4	8.08	N.A.	6.21	2.65	2.88	4.31
C^5a	7.18	5.06	5.78	1.88	2.48	4.71
C^5b	7.17	5.04	5.78	1.87	2.47	4.70
T^6a	7.58	1.49	6.24	2.50	n.o.	n.o.
T^6b	7.56	1.45	6.24	2.50	n.o	n.o
X^7	N.A.	N.A.	N.A.	N.A.	N.A.	N.A.
A^8a	8.42	N.A.	5.94	2.79	2.90	5.02
A^8b	8.41	N.A.	5.93	2.78	2.89	5.01
G^9	7.74	N.A.	5.55	2.63	2.70	4.40
A^{10}	8.10	N.A.	6.21	2.62	2.86	5.02
C^{11}	7.33	5.27	5.94	2.09	2.41	4.75
T^{12}	7.50	1.67	6.22	2.25	2.25	4.54
A^{13}	8.04	N.A.	5.96	2.54	2.66	4.82
G^{14}	7.86	N.A.	5.92	2.66	2.79	4.40
T^{15}	7.25	1.29	6.03	2.19	2.48	4.27
C^{16}	7.45	5.41	5.92	1.93	2.40	4.69
T^{17}a	7.04	0.90	5.93	2.15	2.40	4.26
T^{17}b	7.60	0.90	5.95	2.07	2.36	n.o.
C^{18}a	8.08	6.21	6.62	2.37	2.63	4.42
C^{18}b	8.07	6.20	6.52	2.35	2.54	n.o.
A^{19}a	8.18	N.A.	5.73	2.44	2.71	4.43
A^{19}b	8.16	N.A.	5.70	2.38	2.64	4.39

$G^{20}a$	7.62	N.A.	5.80	2.48	2.70	4.35
$G^{20}b$	7.61	N.A.	5.78	2.46	n.o.	n.o.
T^{21}	7.21	1.23	6.04	2.17	2.51	4.24
C^{22}	7.59	5.58	6.00	2.09	2.48	4.80
T^{23}	7.34	1.73	5.80	1.95	2.35	n.o.
G^{24}	7.94	N.A.	6.17	2.37	2.65	n.o.

Table S2. Chemical shifts (ppm) of exchangeable DNA protons. N.A. = not applicable; n.o. = not observed; o. = overlapped.

Nucleotide	N1H/N3H	N^4H_a	N^4H_b
C^1	N.A.	N.A.	N.A.
A^2	N.A.	N.A.	N.A.
G^3	12.57	N.A.	N.A.
A^4	N.A.	N.A.	N.A.
C^5	N.A.	n.o.	n.o.
T^6a	13.24	N.A.	N.A.
T^6b	13.50	N.A.	N.A.
X^7	N.A.	N.A.	N.A.
A^8	N.A.	N.A.	N.A.
G^9	12.	N.A.	N.A.
A^{10}	N.A.	N.A.	N.A.
C^{11}	N.A.	7.34	8.21
T^{12}	n.o.	N.A.	N.A.
A^{13}	N.A.	N.A.	N.A.
G^{14}	12.74	N.A.	N.A.
T^{15}	13.55	N.A.	N.A.
C^{16}	N.A.	5.78	7.89
T^{17}a	13.52	N.A.	N.A.
T^{17}b	13.45	N.A.	N.A
C^{18}	N.A.	n.o.	n.o.
A^{19}	N.A.	N.A.	N.A.
G^{20}	12.76	N.A.	N.A.
T^{21}	13.65	N.A.	N.A.
C^{22}	N.A.	6.45	8.64
T^{23}	14.15	N.A.	N.A.
G^{24}	n.o.	N.A.	N.A.

Table S3. NOE distance restraints of PIXa-AP conjugate (R_a) DNA duplex used for rMD calculations in AMBER.

Nucleotide 1	Proton	Nucleotide 2	Proton	Lower (Å)	Upper (Å)	Window (Å)
H6	1	H1'	1	2.62	3.815	0.598
H3'	1	H6	1	3.112	4.621	0.754
H2'	1	H1'	1	2.26	3.72	0.73
H2'	1	H6	1	1.862	2.122	0.13
H2'	1	H5	1	2.901	4.154	0.626
H2"	1	H1'	1	1.867	2.136	0.134
H2"	1	H6	1	1.834	3.946	1.036
H8	2	H1'	1	3.449	5.427	0.969
H8	2	H3'	1	3.495	4.116	0.311
H8	2	H2'	1	2.495	3.33	0.417
H8	2	H2"	1	2.276	2.761	0.242
H8	2	H1'	2	3.083	4.391	0.654
H3'	2	H8	2	3.97	5.485	0.577
H2'	2	H8	2	1.877	3.285	0.704
H2"	2	H1'	2	2.046	2.491	0.223
H2"	2	H8	2	1.652	2.969	0.659
H8	3	H1'	2	2.69	3.479	0.395
H8	3	H3'	2	2.486	2.912	0.213
H8	3	H2'	2	2.65	3.623	0.486
H8	3	H2"	2	2.358	3.242	0.442
H8	3	H1'	3	2.452	3.307	0.427
H3'	3	H8	3	3.808	5.081	0.416
H2'	3	H8	3	1.876	2.174	0.149
H2"	3	H8	3	1.927	2.459	0.266
H8	4	H1'	3	2.281	2.719	0.219
H8	4	H2"	3	2.054	2.417	0.182
H8	4	H1'	4	2.15	2.733	0.292
H2"	4	H8	4	1.836	2.274	0.219

H6	5	H1'	4	2.519	3.602	0.542
H6	5	H3'	4	3.242	4.493	0.626
H6	5	H2'	4	2.478	3.249	0.386
H6	5	H2"	4	2.218	2.777	0.279
H6	5	H1'	5	3.285	4.39	0.553
H5	5	H8	4	2.663	3.34	0.338
H3'	5	H6	5	3.229	4.504	0.638
H2'	5	H1'	5	2.528	3.148	0.31
H2'	5	H6	5	2.348	3.602	0.627
H2"	5	H6	5	1.875	2.81	0.467
H6	6	H1'	5	3.481	4.312	0.415
H6	6	H3'	5	3.572	4.938	0.683
H6	6	H2'	5	2.949	3.98	0.516
H6	6	H1'	6	2.977	3.501	0.262
M7	6	H1'	4	5.515	6.873	0.679
M7	6	H1'	5	4.49	6.119	0.814
M7	6	H6	5	3.372	3.931	0.280
M7	6	H1'	6	4.831	6.051	0.610
H2'	6	H1'	6	1.762	2.464	0.351
H1	7	M7	6	3.997	4.367	0.185
H1	7	H2'	6	4.002	4.443	0.220
H3	7	M7	6	5.329	5.947	0.309
H3	7	H4	7	2.709	2.869	0.080
H8	8	H1'	8	3.311	4.523	0.606
H2'	8	H8	8	2.218	2.893	0.337
H2"	8	H8	8	2.531	3.691	0.580
H8	9	H1'	8	2.842	3.148	0.153
H8	9	H3'	8	2.5	2.893	0.197
H8	9	H2"	8	2.812	3.517	0.352
H8	9	H1'	9	2.418	3.446	0.514
H3'	9	H8	9	4.122	6.082	0

H2'	9	H8	9	1.738	2.364	0.313
H2"	9	H8	9	1.789	2.576	0.393
H8	10	H1'	9	2.334	2.715	0.191
H8	10	H2"	9	2.068	2.405	0.168
H8	10	H1'	10	2.232	2.918	0.343
H2"	10	H8	10	1.849	2.19	0.170
H6	11	H1'	10	3.177	3.404	0.114
H6	11	H2'	10	2.375	3.117	0.371
H6	11	H2"	10	2.323	2.692	0.184
H6	11	H1'	11	2.785	3.8	0.508
H5	11	H8	10	3.151	4.365	0.607
H5	11	H2'	10	3.309	4.335	0.493
H5	11	H2"	10	2.553	2.999	0.223
H3'	11	H6	11	3.201	4.665	0.732
H2'	11	H1'	11	2.021	2.617	0.298
H2'	11	H5	11	2.613	3.218	0.303
H2"	11	H6	11	2.107	3.535	0.714
H2"	11	H5	11	3.495	5.259	0.862
H6	12	H1'	11	3.213	3.93	0.359
H6	12	H3'	11	4.637	5.847	0.585
H6	12	H2'	11	2.483	2.955	0.236
H6	12	H2"	11	2.446	3.276	0.415
H6	12	H1'	12	2.841	3.922	0.541
M7	12	H1'	11	4.227	5.072	0.423
M7	12	H6	11	3.183	3.627	0.222
M7	12	H5	11	3.519	3.865	0.173
M7	12	H1'	12	4.673	6.285	0.806
H3'	12	H6	12	2.401	3.653	0.626
H2'	12	H6	12	1.98	2.151	0.085
H2"	12	H6	12	1.963	2.164	0.100
H8	13	H1'	13	2.356	3.001	0.322

H2'	13	H1'	13	1.911	2.342	0.216
H2'	13	H8	13	2.026	2.711	0.343
H2"	13	H8	13	2.37	3.155	0.392
H8	14	H1'	13	2.221	3.608	0.673
H8	14	H2'	13	2.537	3.683	0.553
H8	14	H2"	13	1.923	2.187	0.132
H8	14	H1'	14	2.962	4.093	0.565
H2'	14	H1'	13	1.974	2.204	0.115
H2'	14	H8	14	2.139	2.86	0.360
H2"	14	H8	14	1.846	2.796	0.475
H6	15	H1'	14	3.126	3.648	0.261
H6	15	H3'	14	5.033	6.757	0.642
H6	15	H2'	14	2.771	3.917	0.573
H6	15	H2"	14	2.52	3.125	0.302
H6	15	H1'	15	3.046	4.109	0.532
M7	15	H8	14	3.036	3.368	0.166
M7	15	H1'	15	3.924	4.646	0.361
H2'	15	H6	15	2.096	3.222	0.563
H2"	15	H6	15	1.958	2.805	0.424
H1'	16	M7	15	3.076	3.435	0.180
H6	16	H1'	15	3.097	3.698	0.300
H6	16	H2'	15	2.562	3.858	0.648
H6	16	H2"	15	2.043	2.786	0.371
H6	16	H1'	16	3.072	3.9	0.414
H5	16	H6	15	3.284	4.6	0.658
H5	16	M7	15	3.977	5.179	0.601
H5	16	H2'	15	2.764	4.189	0.713
H5	16	H2"	15	2.703	3.777	0.537
H3'	16	H6	16	3.203	4.263	0.530
H2'	16	H1'	16	3.236	5.196	0.000
H2'	16	H6	16	2.186	3.134	0.474

H2'	16	H5	16	3.167	4.366	0.599
H2"	16	H6	16	2.415	3.513	0.549
H2"	16	H5	16	3.139	4.318	0.589
H1'	17	M7	15	4.39	4.928	0.269
H6	17	H1	7	5.819	6.556	0.368
H6	17	H3	7	4.185	5.103	0.459
H6	17	H1'	16	2.898	5.086	1.094
H6	17	H3'	16	4.805	5.744	0.35
H6	17	H2"	16	1.915	2.361	0.223
H6	17	H1'	17	3.099	4.803	0.852
M7	17	H4	7	4.001	4.435	0.217
M7	17	H1	7	4.141	4.517	0.188
M7	17	H3	7	4.097	4.608	0.256
M7	17	H1'	15	4.477	5.437	0.48
M7	17	H1'	16	4.197	5.245	0.524
M7	17	H6	16	3.072	3.624	0.276
M7	17	H5	16	3.723	4.453	0.365
M7	17	H1'	17	4.187	5.279	0.546
H3'	17	H6	17	5.491	7.829	0.689
H2'	17	H3	7	3.773	5.395	0.811
H2'	17	H6	17	2.132	4.084	0.976
H2"	17	H3	7	3.377	4.085	0.354
H2"	17	H6	17	2.028	2.663	0.317
H1'	18	H3	7	5.459	6.138	0.339
H6	18	H1'	18	3.589	5.04	0.725
H5	18	H6	17	4.321	6.126	0.762
H2'	18	H1'	18	2.413	3.61	0.599
H2'	18	H6	18	1.949	2.621	0.336
H2"	18	H1'	18	2.36	3.436	0.538
H8	19	H1'	18	4.154	4.58	0.213
H8	19	H3'	18	2.858	3.304	0.223

H8	19	H2'	18	3.454	4.63	0.588
H8	19	H2"	18	3.149	4.479	0.665
H8	19	H1'	19	3.396	4.207	0.405
H3'	19	H8	19	2.869	3.531	0.331
H2'	19	H1'	19	2.488	3.717	0.614
H2'	19	H8	19	2.35	2.969	0.309
H2"	19	H1'	19	2.233	3.098	0.433
H2"	19	H8	19	2.75	3.888	0.569
H8	20	H1'	19	3.748	4.743	0.498
H8	20	H2'	19	3.049	4.157	0.454
H8	20	H2"	19	2.071	2.571	0.25
H8	20	H1'	20	3.572	4.923	0.675
H2'	20	H8	20	2.621	3.465	0.422
H2"	20	H8	20	2.133	2.8	0.333
H6	21	H1'	20	2.94	4.105	0.582
H6	21	H2'	20	1.873	2.395	0.261
H6	21	H2"	20	2.481	3.721	0.6
H6	21	H1'	21	2.913	4.248	0.667
M7	21	H8	20	3.535	4.136	0.3
M7	21	H1'	21	4.03	5.04	0.505
H2'	21	H6	21	1.969	2.621	0.326
H2"	21	H6	21	2.126	2.73	0.302
H6	22	H1'	21	3.113	4.236	0.561
H6	22	H2'	21	2.323	2.481	0.079
H6	22	H2"	21	2.23	2.463	0.117
H6	22	H1'	22	2.782	4.027	0.622
H5	22	H6	21	3.129	3.705	0.288
H5	22	M7	21	3.587	4.252	0.332
H3'	22	H6	22	5.478	6.598	0.26
H2'	22	H1'	22	2.109	3.25	0.57
H2'	22	H6	22	2.214	2.433	0.11

H2"	22	H1'	22	1.993	2.844	0.425
H2"	22	H6	22	2.217	2.51	0.146
H6	23	H1'	22	2.928	3.926	0.499
H6	23	H2"	22	2.139	3.353	0.607
H6	23	H1'	23	3.04	3.86	0.41
M7	23	H1'	21	4.982	6.693	0.856
M7	23	H1'	22	3.737	5.735	0.999
M7	23	H6	22	3.094	3.468	0.187
M7	23	H5	22	3.616	4.066	0.225
M7	23	H1'	23	3.698	4.922	0.612
H2'	23	H1'	23	2.346	3.292	0.473
H2'	23	H6	23	1.882	2.243	0.18
H2"	23	H1'	23	2.005	2.347	0.171
H2"	23	H6	23	2.159	2.861	0.351
H8	24	H1'	23	2.586	4.396	0.905
H8	24	M7	23	3.976	5.751	0.887
H8	24	H2'	23	2.136	2.627	0.246
H8	24	H2"	23	2.133	2.63	0.249
H8	24	H1'	24	2.75	4.509	0.879
H2'	24	H1'	24	1.819	2.215	0.198
H2'	24	H8	24	2.029	2.517	0.244
H2"	24	H1'	24	2.066	3.279	0.607
H2"	24	H8	24	1.954	2.13	0.088
H2"	24	H8	24	1.954	2.13	0.088

Table S4. NOE distance restraints of PIXb-AP conjugate (R_b) DNA duplex used for rMD calculations in AMBER.

Nucleotie 1	Proton	Nucleotide 2	Proton	Lower (Å)	Upper (Å)	Window (Å)
H6	1	H1'	1	2.122	2.327	0.103
H3'	1	H6	1	2.346	2.574	0.114
H2'	1	H1'	1	3.123	4.166	0.522
H2'	1	H6	1	2.364	2.517	0.076
H2'	1	H5	1	2.389	2.611	0.111
H2"	1	H1'	1	2.252	2.374	0.061
H2"	1	H6	1	3.395	5.192	0.858
H8	2	H3'	1	2.99	3.375	0.192
H8	2	H2'	1	3.704	5.057	0.677
H8	2	H2"	1	4.24	5.356	0.558
H8	2	H1'	2	2.058	2.404	0.173
H3'	2	H8	2	2.567	3.079	0.256
H2'	2	H8	2	2.086	2.336	0.125
H2"	2	H1'	2	2.154	2.376	0.111
H2"	2	H8	2	2.704	3.34	0.318
H8	3	H1'	2	2.782	3.326	0.272
H8	3	H3'	2	2.818	3.331	0.257
H8	3	H2'	2	3.23	4.045	0.408
H8	3	H2"	2	3.951	5.43	0.739
H3'	3	H8	3	2.697	2.812	0.058
H2'	3	H8	3	2.403	2.703	0.15
H2"	3	H8	3	2.368	2.586	0.109
H8	4	H2"	3	2.532	3.139	0.304
H2"	4	H8	4	2.318	2.482	0.082
H6	5	H1'	4	3.025	3.823	0.399
H6	5	H3'	4	2.732	3.025	0.147
H6	5	H2'	4	2.33	2.606	0.138
H6	5	H2"	4	2.714	4.348	0.817

H6	5	H1'	5	2.22	2.558	0.169
H3'	5	H6	5	2.543	2.756	0.107
H2'	5	H1'	5	2.128	2.295	0.083
H2'	5	H6	5	2.571	3.468	0.448
H2''	5	H6	5	2.228	2.435	0.103
H6	6	H1'	5	2.347	3.128	0.39
H6	6	H3'	5	2.402	2.831	0.214
H6	6	H2'	5	2.394	2.978	0.292
H6	6	H1'	6	3.018	3.122	0.052
M7	6	H1'	5	3.753	4.244	0.245
M7	6	H6	5	4.086	4.534	0.224
M7	6	H1'	6	4.509	5.045	0.268
H2'	6	H1'	6	2.231	2.421	0.095
H4	7	M7	6	4.092	4.416	0.162
H1	7	M7	6	6.85	7.625	0.387
H3	7	M7	6	4.954	5.468	0.257
H3	7	H4	7	3.255	3.571	0.158
H8	8	H1'	8	3.168	4.729	0.78
H2'	8	H8	8	2.142	2.38	0.119
H2''	8	H8	8	2.08	2.221	0.07
H8	9	H1'	8	3.62	4.013	0.196
H8	9	H3'	8	2.775	3.244	0.234
H8	9	H2''	8	3.523	5.111	0.794
H3'	9	H8	9	2.713	2.817	0.052
H2'	9	H8	9	2.335	2.629	0.147
H2''	9	H8	9	2.38	2.659	0.14
H8	10	H2''	9	2.397	2.807	0.205
H2''	10	H8	10	2.392	2.749	0.179
H6	11	H1'	10	3.192	3.448	0.128
H6	11	H3'	10	2.901	3.065	0.082
H6	11	H2'	10	2.188	2.492	0.152

H6	11	H2"	10	2.951	3.852	0.451
H6	11	H1'	11	3.302	4.727	0.713
H5	11	H8	10	2.234	2.56	0.163
H5	11	H2'	10	2.294	3.499	0.603
H5	11	H2"	10	2.163	2.937	0.387
H3'	11	H6	11	2.417	2.636	0.11
H2'	11	H1'	11	2.083	2.247	0.082
H2'	11	H5	11	2.444	4.051	0.803
H2"	11	H6	11	2.238	2.684	0.223
H2"	11	H5	11	2.288	4.004	0.858
H6	12	H1'	11	2.864	3.244	0.19
H6	12	H3'	11	3.734	4.085	0.175
H6	12	H2'	11	3.524	5.02	0.748
H6	12	H2"	11	3.514	4.798	0.642
H6	12	H1'	12	2.822	2.96	0.069
M7	12	H1'	11	3.327	4.247	0.46
M7	12	H6	11	4.614	6.214	0.68
M7	12	H5	11	2.69	2.977	0.143
M7	12	H1'	12	3.193	3.767	0.287
H3'	12	H6	12	2.79	2.891	0.05
H2'	12	H6	12	2.407	2.766	0.179
H2"	12	H6	12	2.502	2.821	0.16
H8	13	H1'	13	2.271	2.758	0.243
H3'	13	H8	13	2.748	3.604	0.428
H2'	13	H1'	13	2.546	3.247	0.351
H2'	13	H8	13	2.053	2.326	0.136
H2"	13	H8	13	2.153	2.469	0.158
H8	14	H1'	13	2.159	2.478	0.16
H8	14	H3'	13	3.034	3.551	0.258
H8	14	H2'	13	3.106	4.401	0.608
H8	14	H2"	13	2.479	2.875	0.198

H8	14	H1'	14	3.09	3.742	0.326
H3'	14	H8	14	2.841	3.237	0.198
H2'	14	H1'	13	2.405	2.674	0.135
H2'	14	H8	14	2.651	3.164	0.257
H2"	14	H8	14	2.287	2.533	0.123
H6	15	H1'	14	3.231	3.913	0.341
H6	15	H3'	14	3.217	3.697	0.24
H6	15	H2'	14	3.384	5.07	0.843
H6	15	H2"	14	2.426	2.975	0.274
H6	15	H1'	15	2.935	3.173	0.119
M7	15	H8	14	3.989	4.574	0.293
M7	15	H1'	15	2.464	3.042	0.289
H3'	15	H6	15	2.722	2.874	0.076
H2'	15	H6	15	2.204	2.448	0.122
H2"	15	H6	15	2.338	2.871	0.267
H1'	16	M7	15	1.769	2.121	0.176
H6	16	H1'	15	2.963	3.164	0.1
H6	16	H3'	15	3.171	3.96	0.394
H6	16	H2'	15	2.437	4.084	0.823
H6	16	H2"	15	2.252	3.4	0.574
H6	16	H1'	16	2.15	2.545	0.198
H5	16	H6	15	2.28	2.719	0.219
H5	16	M7	15	2.417	3.112	0.348
H5	16	H2'	15	2.124	3.646	0.761
H5	16	H2"	15	2.248	3.632	0.692
H3'	16	H6	16	2.323	2.549	0.113
H2'	16	H1'	16	1.965	2.873	0.454
H2'	16	H6	16	2.241	2.69	0.224
H2'	16	H5	16	2.5	3.402	0.451
H2"	16	H6	16	2.215	2.589	0.187
H2"	16	H5	16	2.62	3.715	0.547

H1'	17	H1	7	5.302	6.397	0.548
H1'	17	M7	15	3.159	3.534	0.187
H6	17	H1'	16	2.861	3.602	0.371
H6	17	H3'	16	3.436	4.763	0.663
H6	17	H2'	16	2.599	2.952	0.176
H6	17	H2"	16	2.385	2.821	0.218
H6	17	H1'	17	2.948	3.238	0.145
M7	17	H1	7	5.028	5.574	0.273
M7	17	H3	7	5.077	5.646	0.285
M7	17	H1'	15	3.025	3.531	0.253
M7	17	H1'	16	2.63	3.036	0.203
M7	17	H5	16	2.572	2.908	0.168
M7	17	H1'	17	2.671	3.163	0.246
H3'	17	H6	17	3.241	3.707	0.233
H2'	17	H1	7	3.954	5.361	0.704
H2'	17	H6	17	2.214	2.37	0.078
H2"	17	H1	7	4.084	5.206	0.561
H2"	17	H6	17	2.398	2.667	0.135
H6	18	H1'	17	4.011	5.971	0.000
H6	18	H2'	17	2.333	3.164	0.416
H6	18	H1'	18	2.651	3.694	0.521
H5	18	H6	17	4.14	5.548	0.704
H3'	18	H6	18	2.659	4.004	0.673
H2'	18	H1'	18	2.028	3.073	0.522
H2'	18	H6	18	1.865	2.169	0.152
H2"	18	H1'	18	1.844	2.336	0.246
H8	19	H1'	18	2.752	3.645	0.446
H8	19	H3'	18	1.958	2.231	0.136
H8	19	H2'	18	2.592	3.441	0.425
H8	19	H1'	19	2.147	2.704	0.279
H3'	19	H8	19	1.943	2.17	0.113

H2'	19	H1'	19	2.165	2.339	0.087
H2'	19	H8	19	2.342	3.906	0.782
H2"	19	H1'	19	2.258	2.442	0.092
H2"	19	H8	19	2.174	2.679	0.253
H8	20	H1'	19	3.022	4.513	0.746
H8	20	H3'	19	3.327	5.024	0.829
H8	20	H2"	19	1.909	2.133	0.112
H8	20	H1'	20	3.788	4.573	0.373
H3'	20	H8	20	3.507	4.803	0.648
H2'	20	H8	20	2.272	3.334	0.531
H2"	20	H8	20	1.918	2.217	0.149
H6	21	H1'	20	3.052	3.755	0.351
H6	21	H2'	20	2.378	2.685	0.153
H6	21	H2"	20	2.316	2.829	0.257
H6	21	H1'	21	3.046	3.261	0.108
M7	21	H8	20	4.153	5.609	0.728
M7	21	H1'	21	2.689	3.156	0.233
H3'	21	H6	21	2.786	2.957	0.085
H2'	21	H6	21	2.309	2.527	0.109
H2"	21	H6	21	2.618	3.589	0.485
H6	22	H1'	21	3.202	3.496	0.147
H6	22	H2'	21	2.582	3.199	0.309
H6	22	H2"	21	2.669	2.968	0.15
H6	22	H1'	22	2.139	2.403	0.132
H5	22	H6	21	3.703	4.269	0.283
H5	22	M7	21	2.173	2.689	0.258
H3'	22	H6	22	3.125	3.37	0.122
H2'	22	H1'	22	2.123	2.283	0.08
H2'	22	H6	22	2.872	3.237	0.182
H2"	22	H1'	22	2.238	3.25	0.506
H2"	22	H6	22	2.94	3.594	0.327

H6	23	H1'	22	2.516	3.274	0.379
H6	23	H2''	22	2.998	4.179	0.591
H6	23	H1'	23	2.04	2.278	0.119
M7	23	H1'	21	3.405	3.865	0.23
M7	23	H1'	22	2.681	3.154	0.237
M7	23	H6	22	4.864	6.803	0.95
M7	23	H5	22	2.553	2.949	0.198
M7	23	H1'	23	2.79	4.063	0.636
H2'	23	H1'	23	2.696	3.822	0.543
H2'	23	H6	23	2.185	2.447	0.131
H2''	23	H1'	23	2.142	2.691	0.275
H2''	23	H6	23	2.951	3.925	0.467
H8	24	H1'	23	2.139	2.387	0.124
H8	24	H2'	23	3.048	3.382	0.167
H8	24	H2''	23	2.947	4.207	0.63
H8	24	H1'	24	1.977	2.158	0.09
H2'	24	H1'	24	2.15	2.572	0.211
H2'	24	H8	24	2.446	3.749	0.572
H2''	24	H1'	24	2.292	3.673	0.67
H2''	24	H8	24	2.188	2.846	0.329
H2''	24	H8	24	2.188	2.846	0.329

Table S5. Selected crystal data, X-ray data collection and refinement parameters.

**Oligonucleotide**	Modified 12-mer
Data Collection	
Oligonucleotide sequence	5′-d(CGCXAATT(Br5C)GCG)-3′[a]
Space group	$P6_422$
Unit cell constants: *a*, *b*, *c* [Å]	95.92, 95.92, 73.12
Unit cell constants: α, β, γ [°]	90.0, 90.0, 120.0
Resolution [Å]	27.71-1.49 (1.58-1.51)[b]
No. of unique reflections	7,367
Completeness (outer shell) [%]	99.9 (100.0)
R-merge (outer shell)	0.131 (1.212)
R-pim (outer shell)	0.022 (0.198)
I/σ(I) (outer shell)	42.00 (2.00)
Redundancy	37.5 (37.9)
Refinement	
Resolution[b]	27.71-2.49 (2.56-2.49)
Number of unique reflections	6,740 (487)
R-work	0.245 (0.620)
R-free	0.262 (0.483)
No. of nucleotide-atoms	506
No. of waters/ethylene glycol	27/4
R.m.s. deviations bonds [Å]	0.010
R.m.s. deviations angles [°]	1.38
Avg. B-factor, nucleotide atoms [Å^2]	49.43[b]
Avg. B-factor, H_2O/ethylene glycol [Å^2]	77.38/36.49

PDB entry code	7U38

[a]X is PIX-AP and Br5C is 5-Bromo-2′-deoxycytidine

[b]Outermost shell in parentheses

Supporting Figures

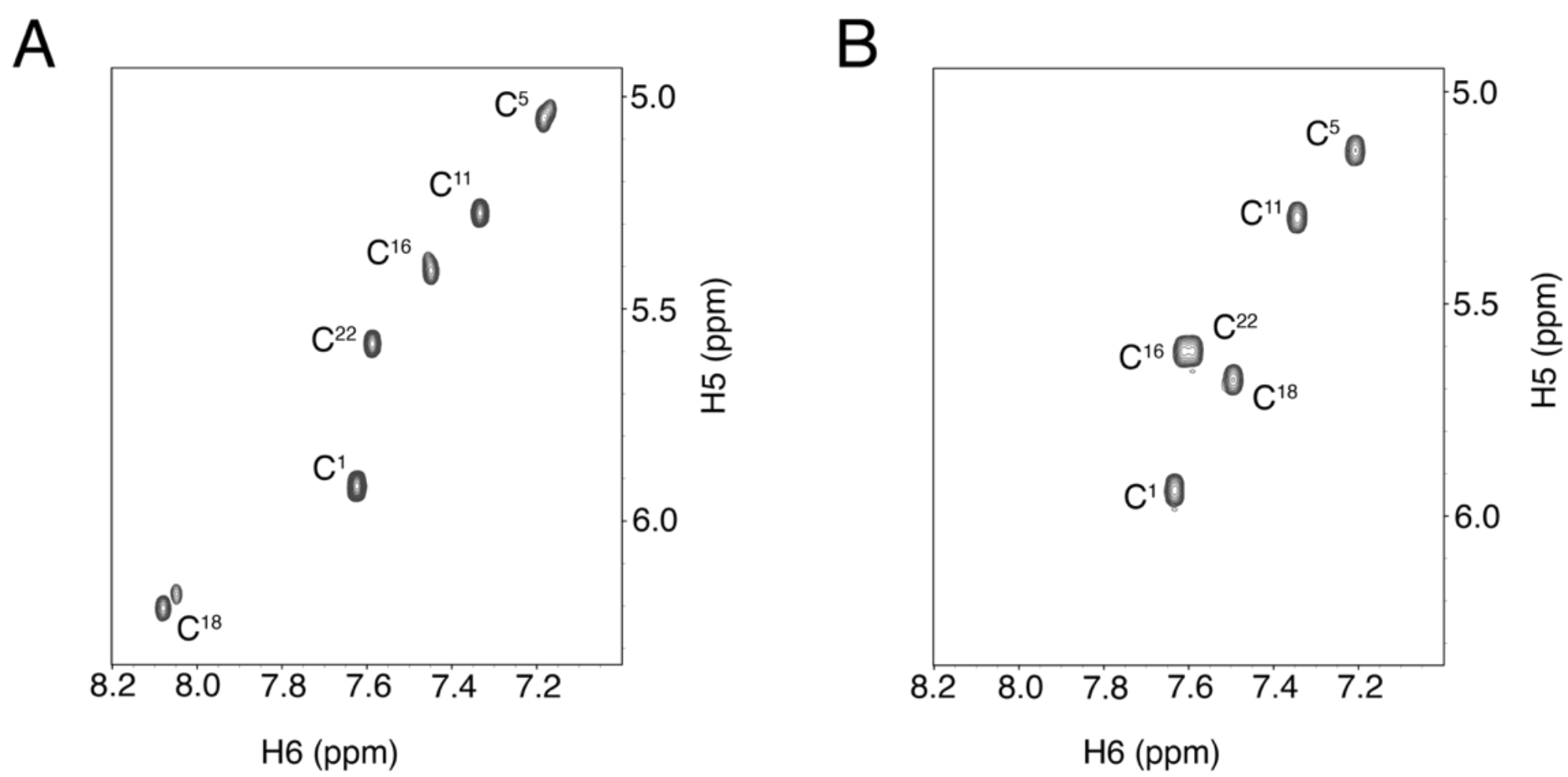

Figure S1. Two-dimensional COSY spectra of PIX-AP conjugate duplex and unmodified duplex. **A.** COSY spectrum of 5´-d($C^1A^2G^3A^4C^5T^6X^7A^8G^9A^{10}C^{11}T^{12}$)-3´:5´-d($A^{13}G^{14}T^{15}C^{16}T^{17}C^{18}A^{19}G^{20}T^{21}C^{22}T^{23}G^{24}$)-3´ (X = PIX-AP) showing scalar couplings between cytosine H5 and H6 protons. **B.** COSY spectrum of 5´-d($C^1A^2G^3A^4C^5T^6X^7A^8G^9A^{10}C^{11}T^{12}$)-3´ duplex. The spectra were acquired at 900 MHz and 288 K.

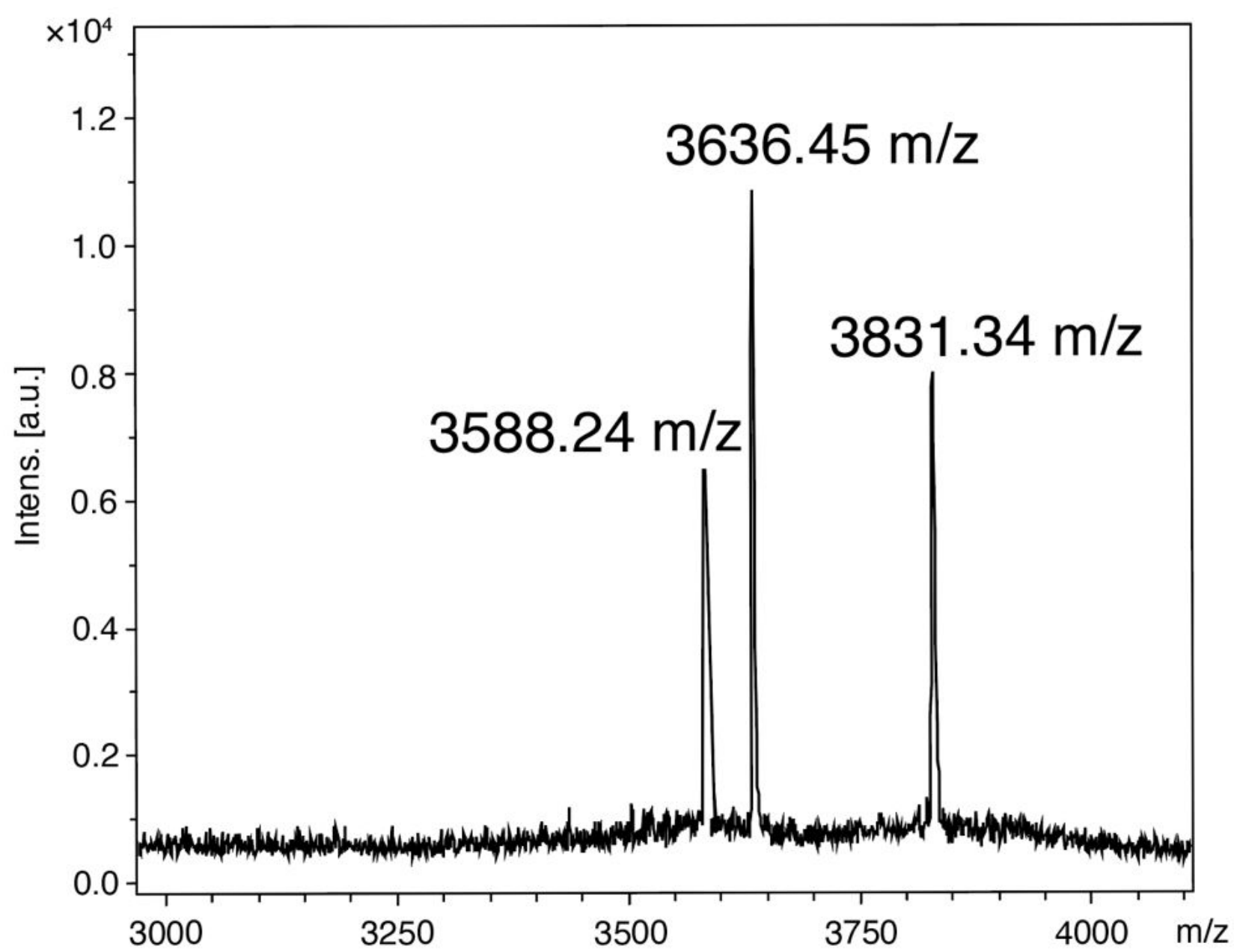

Figure S2. MALDI-TOF mass spectrum of the methoxyamine (MX)-quenched reaction. The peak at 3588.24 m/z represents the expected mass of the MX-containing strand, while the peaks at 3636.45 m/z and 3831.34 m/z represents the expected mass of the corresponding complementary strand and PIX-AP conjugate strand, respectively.

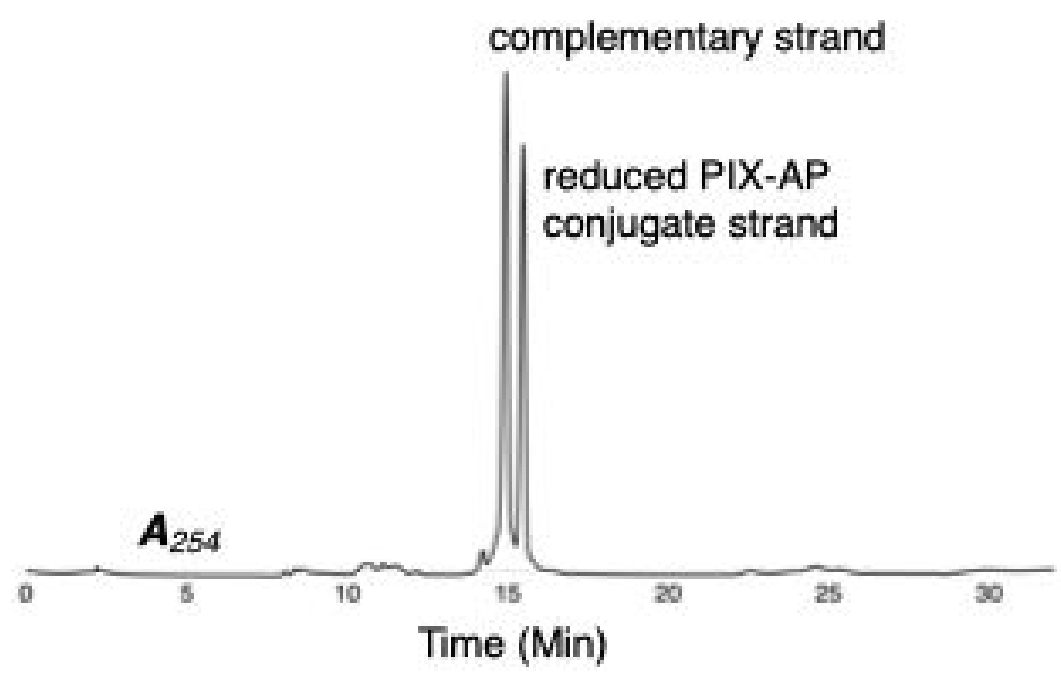

Figure S3. HPLC-UV chromatogram of the reaction of the AP-containing oligonucleotide with PIX reduced with $NaB(CN)H_3$ at 60 °C analyzed after 1 h.

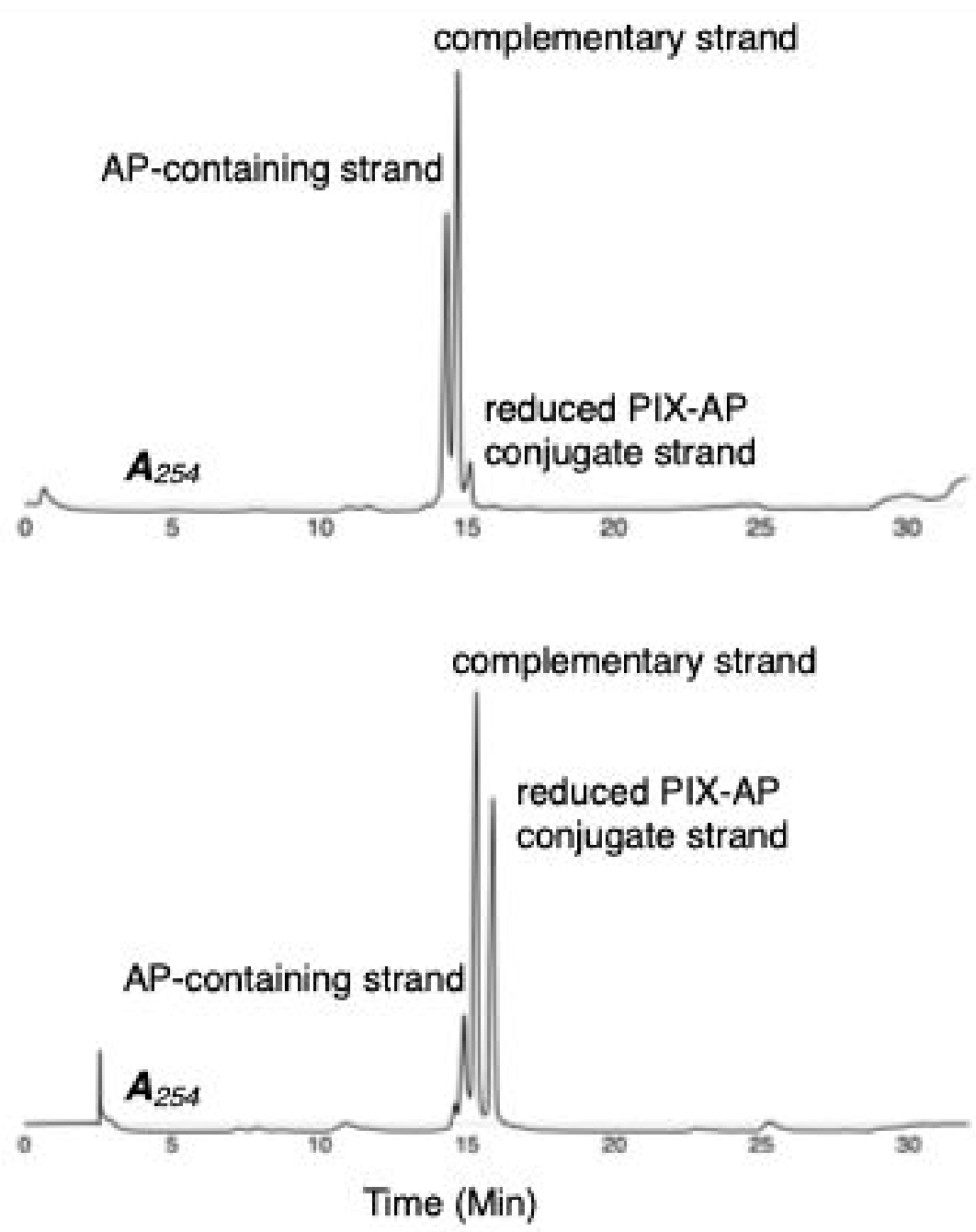

Figure S4. HPLC-UV chromatogram of the reaction of the AP-containing oligonucleotide with PIX reduced with $NaB(CN)H_3$ analyzed at 0 °C (top) and 35 °C (bottom) after 1 h.

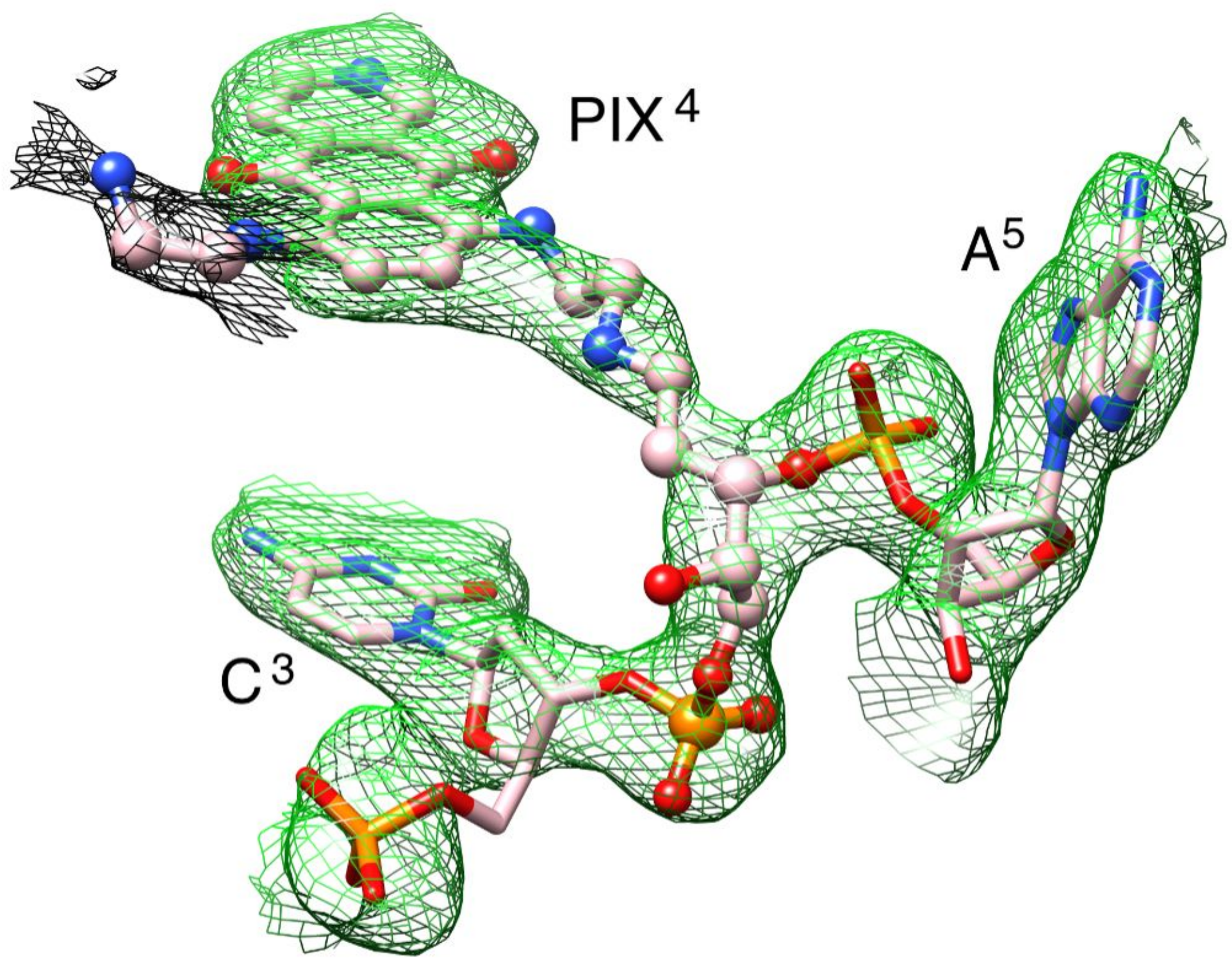

Figure S5. Final Fourier $2F_o$-F_c sum electron density (green meshwork) around the C^3-PIX-A^5 base steps drawn at ~1.1 σ threshold. The electron density around one of the diamino ethyl arms is weaker and therefore shown at ~0.8 σ threshold (grey meshwork). PIX-AP atoms are depicted in ball and stick mode. Atoms are colored salmon, red, blue and orange for carbon, oxygen, nitrogen, and phosphorus, respectively.

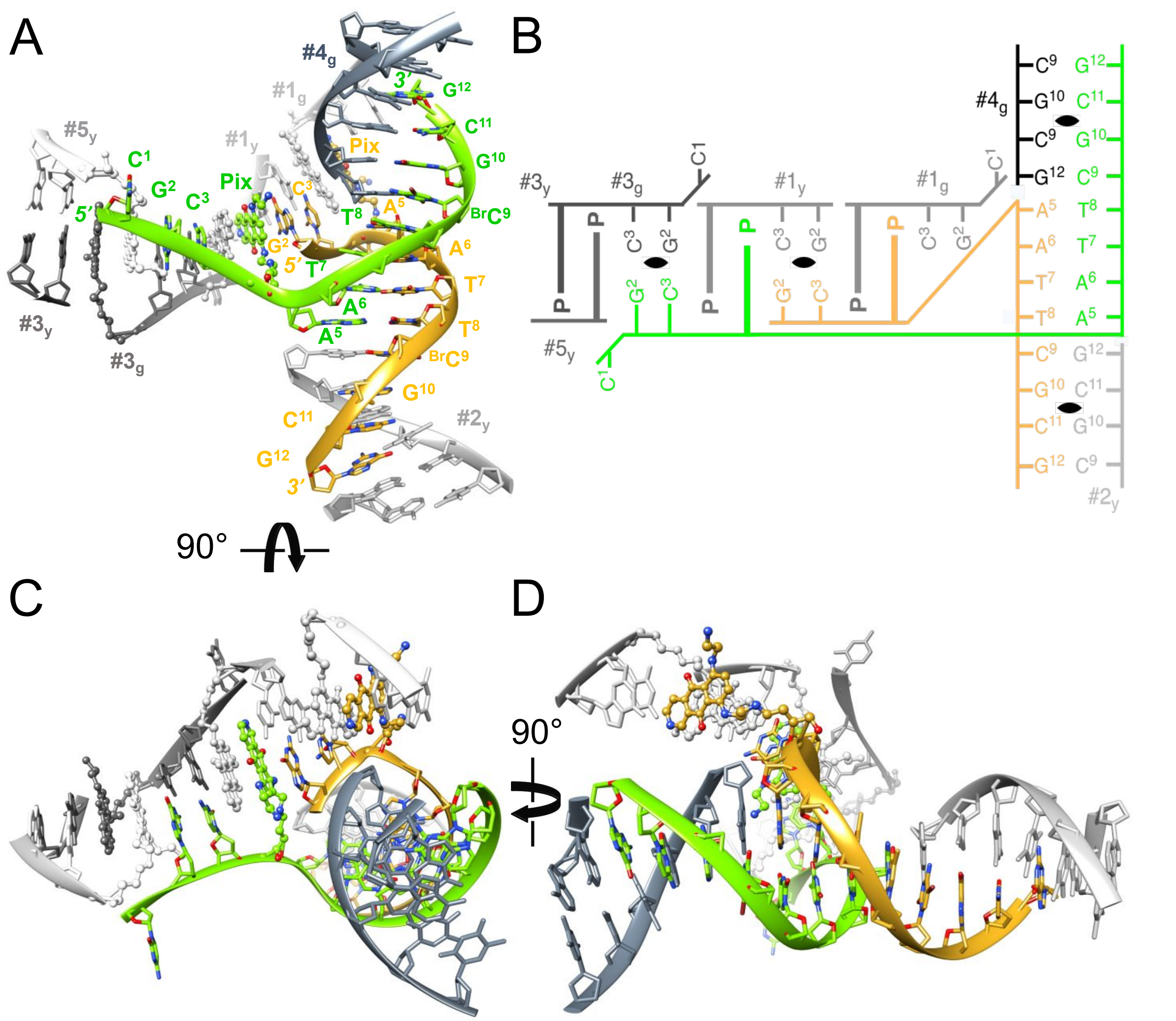

Figure S6. Lattice interactions in the crystal structure of a modified DDD with a PIX-AP conjugate. **A.** A kink in the 12-mer strands between the PIX moiety and the adjacent adenosine results in looped-out tetramers and gives rise to an infinite duplex that consists of alternating CGCG G-tracts and AATT A-tracts and roughly perpendicular crossovers that consists of alternating CG dimers and stacked PIX moieties. Symmetry-related yellow and green strands are marked $\#x_y$ and $\#x_g$, respectively, residues of the parent yellow and green strands are labeled, and the positions of crystallographic dyads are indicated. The 5′-terminal C^1 nucleosides of yellow strands are not visible in the electron density and 5′ -terminal C^1 nucleosides of green strands are extruded from the connector duplex. **B.** Schematic drawing of the lattice interactions shown in panel A with positions of crystallographic twofold rotation axes

indicated. **C.** The crossover of duplexes depicted in panel A rotated by 90° around the horizontal axis. **D.** The crossover of duplexes depicted in panel C rotated by 90° around the vertical axis.

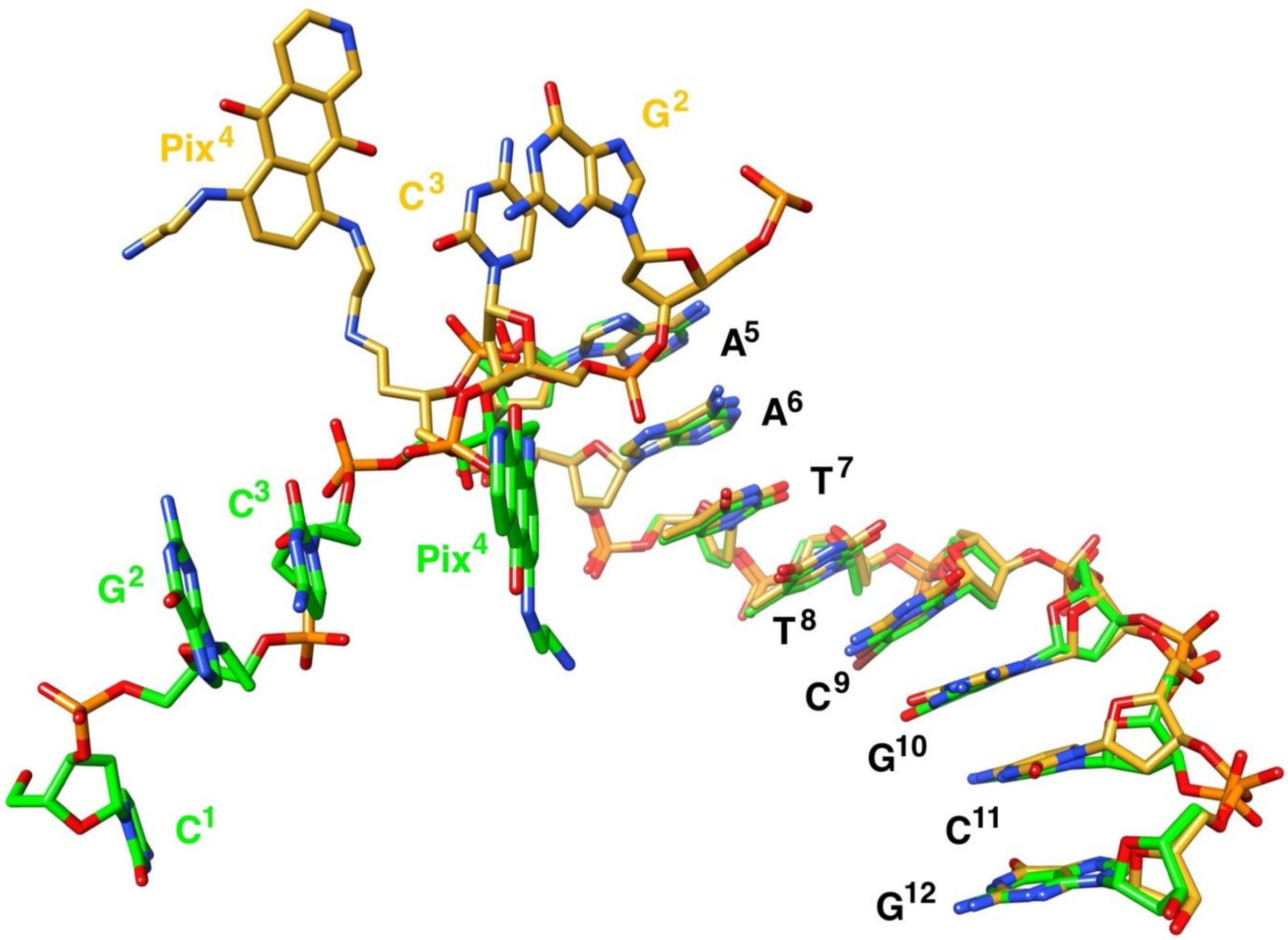

Figure S7. Superimposition of yellow and green DDD strands with PIX-AP conjugates. The resolution of the structure does not allow us to distinguish between nitrogen and carbon in the aza-anthracene-9,10-dione. As well the immediate environment of the ring in the crystal lattice is devoid of H-bond donors or acceptors that would allow a distinction between N and C-H. Hence N positions shown are arbitrarily chosen.

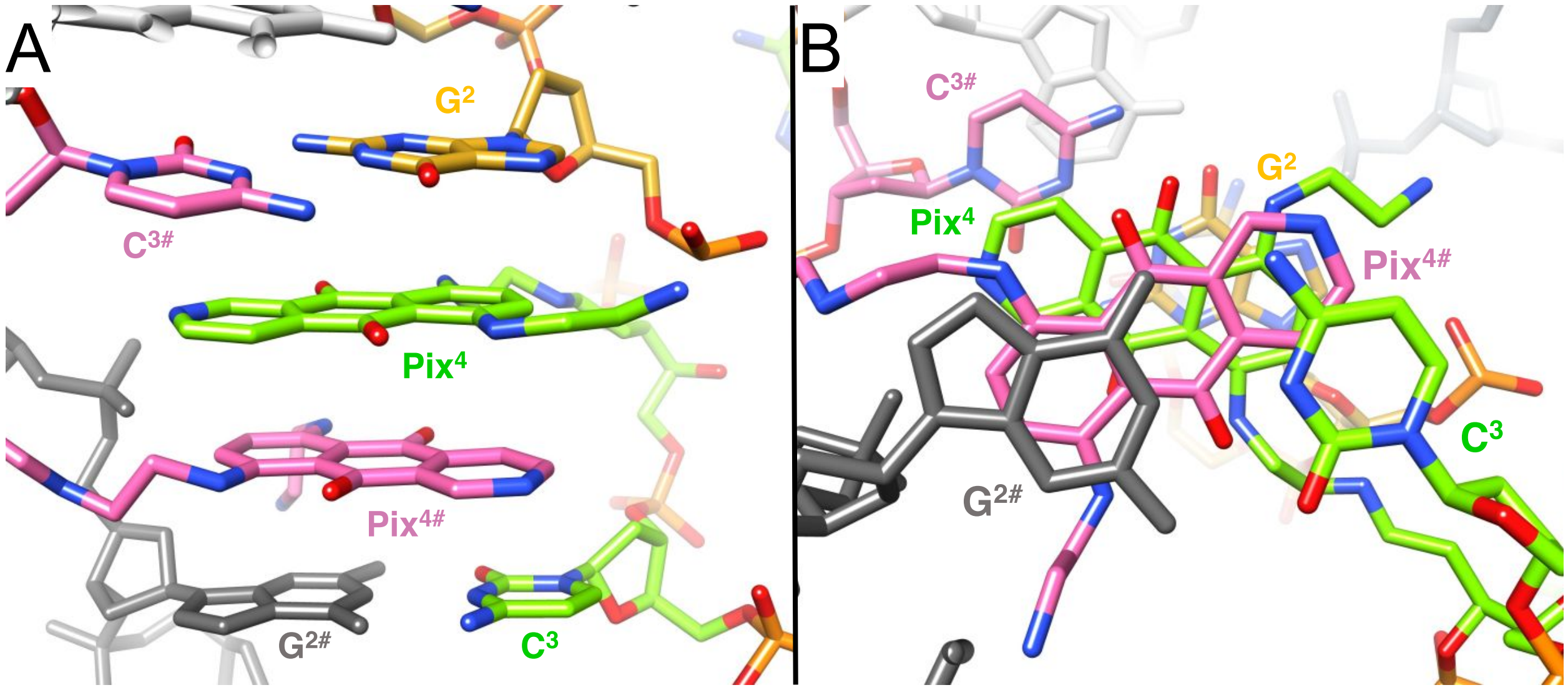

Figure S8. Stacked PIX moieties alternating with CG dimers in the connector duplex. **A.** View into the pseudo major groove. **B.** View approximately along the normal to PIX and base planes. PIX moieties and nucleotides from the parent strands (yellow and green) and contributed by symmetry mates are colored differently.
